# Machine Learning-Driven Simulations of the SARS-CoV‑2 Fitness Landscape from Deep Mutational Scanning Experiments

**DOI:** 10.1021/acs.jcim.6c00332

**Published:** 2026-05-06

**Authors:** Aleksander E. P. Durumeric, Sean McCarty, Jay Smith, Jonas Köhler, Katarina Elez, Lluís Raich, Patricia A. Suriana, Terra Sztain

**Affiliations:** † Department of Mathematics and Computer Science, 9166 Freie Universität Berlin, Arnimallee 12, Berlin 14195, Germany; ‡ Institute for Theoretical Physics, Heidelberg University, Heidelberg 69120, Germany; § Department of Medicinal Chemistry, 1259 University of Michigan, 428 Church St., Ann Arbor, Michigan 48109, United States; ∥ Department of Computer Science, 6429 Stanford University, 353 Jane Stanford Way, Stanford, California 94305, United States; ⊥ Department of Biophysics, 1259 University of Michigan, 930 N University Ave, Ann Arbor, Michigan 48109, United States

## Abstract

Predicting protein variant effects is a key challenge in preparing for pathogenic viral strains, understanding mutation-linked diseases, and designing new proteins. Protein sequence–structure–function relationships are difficult to model due to complex allosteric and epistatic effects. To investigate efficient modeling strategies, we trained supervised machine learning (ML) models with deep mutational scanning (DMS) libraries of SARS-CoV-2 receptor binding domain (RBD) sequences labeled with angiotensin converting enzyme 2 (ACE2) binding affinity. These models demonstrate superior performance predicting combinatorial mutation effects compared to adding or averaging the effects of point mutations and exhibit strong extrapolative performance ranking omicron variants when training only near wild type (WT) variants. We characterize the RBD fitness landscape by combining ML with Markov Chain Monte Carlo simulations to predict evolutionary patterns from the WT sequence. These generate comparable sequence profiles to high-fitness sequences in DMS data and predict mutations in unseen omicron variants. These models provide insight into the relationship between RBD sequence elements and offer a new perspective on the use of DMS to predict emerging viral strains, which we anticipate will be applicable to other evolutionary prediction tasks. To facilitate application and future development of this strategy, we introduce Mavenets: https://github.com/SztainLab/mavenets.

## Graphical abstract

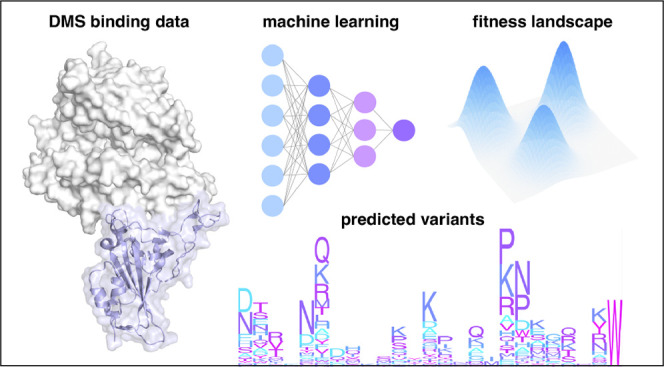

## Introduction

The SARS-CoV-2 virus, which led to the COVID-19 pandemic, continues to evolve, accumulating mutations that evade immune responses and therapies.^1–4^ Preparation for future variants remains challenging due to the large number of possible mutations that could cause virulent strains. Most problematic mutations occur in the receptor binding domain (RBD) of the spike protein, which binds angiotensin converting enzyme 2 (ACE2) on host cells as the first point of contact for viral infection.^5^ Starr et al. performed deep mutational scanning (DMS) on the spike RBD in 2020,^6^ providing sequence-function information characterizing the effect of 10^5^ RBD sequence variants on ACE2 binding, a strong indicator of viral fitness.^7–9^ Additional experiments initialized from the B1.351 variant in 2021 ^10^ and omicron BA.1 and BA.2 in 2022 ^11^ were subsequently carried out using library generation methods optimized to produce saturating point mutations at each position, each characterizing a smaller number (10^4^) of additional sequences. However, these studies are inherently unable to provide comprehensive information on all possible mutations (e.g., 20^200^ ≈ 10^260^ for a 200 residue protein). Furthermore, the reported fitness estimate for any single sequence is noisy, impeding the isolation of important mutants and hampering the understanding of individual mutations.

The ability to computationally predict the effect of any mutation on viral fitness would allow scientists to quickly isolate which mutations might be problematic in the future. However, this dependence is difficult to model. Due to the complex nature of protein sequence-function relationships, the effect of combined mutations cannot be accurately estimated by adding or averaging the effects of single mutations.^12–15^ Machine learning (ML) has shown substantial success modeling complex interdependencies;^16^ for example, ML has been transformative for molecular biology, with approaches such as Alphafold predicting sequence-structure relationships.^17–21^ Similarly, approaches based on protein language models (PLMs), such as ESM^18^ and ProtT5,^22^ have learned generalizable sequence–function relationships by leveraging massive protein sequence data sets. Additionally, coevolutionary models, including EVE^23^ and GEMME,^24^ have used information from multiple sequence alignments to infer mutational effects. More recently, hybrid approaches that integrate PLM, coevolutionary or explicit structural representations have shown improvements for specialized protein prediction tasks.^25–29^ While these approaches are designed for broad transferability, their generalization to highly specialized or out-of-distribution regimes remains variable,^30,31^ often necessitating domain-specific adaptation via fine-tuning.^32–35^ Furthermore, the substantial computational resources required for these large models may pose practical constraints in some applications.

For well-defined, task-specific applications, dedicated models trained on focused data can be advantageous. The development of specialized protein fitness predictors has greatly benefited from high-throughput data generated by multiplex assays of variant effect (MAVE),^36^ such as deep mutational scanning (DMS).^37^ For example, Gelman et al. demonstrated that neural networks can learn sequence-function relationships from DMS data, capture nonlinear effects, and guide protein design.^38^ Tareen et al. introduced MAVE-NN for modeling genotype-phenotype relationships from MAVE data using a combination of statistical and neural network methods.^39^ Faure et al. developed MoCHI, a flexible framework that combines neural networks with interpretable statistical metrics, and uniquely enables higher-order epistatic modeling and integration of multimodal phenotypic data.^40^

These approaches have been applied to learn SARS-CoV-2 RBD fitness. Taft et al. described an approach for “deep mutational learning,” which integrates DMS data to rationally design mutagenesis libraries targeting specific regions of interest.^15^ Machine learning classifiers trained on these combinatorial libraries enabled accurate characterization of RBD variants as binders or nonbinders to ACE2 and various therapeutic antibodies. Importantly, they found that point mutations were not additive at higher edit distances from wild type (WT), and combinatorial libraries were critical to model performance. As noted by the authors, this study focuses only on combinatorial mutations of select sites known to mutate in variants of concern (VOC), limiting the predictive scope.

Many others have developed predictive models of SARS-CoV-2 spike fitness;^41–51^ however, very few have focused on the capacity for early prediction when minimal sequence and experimental data are available from early strains.^52^ In this work, we train RBD fitness predictors with DMS libraries and evaluate their ability to predict the fitness of unseen sequences. Accuracy is characterized using held-out data from the DMS experiment used for training, data from fully held-out DMS experiments, and by sampling the fitness landscapes defined using the output of the fitness predictor as an energy function with Markov chain Monte Carlo (MCMC).

Models trained on a large combinatorial DMS library (10^5^) initiated from the WT sequence are found to accurately characterize trends in held-out data from the experiment used for training and accurately predict patterns present in held-out DMS experiments, including sequences with much greater edit distances than found in training, such as omicron strains. Models trained on smaller libraries focused on attaining saturating point mutagenesis showed much worse predictive capacity. Fitness landscape simulations are assessed by their the capacity to predict the evolution of mutations observed in the population. The resulting sequence profiles prioritize hotspot residue positions and amino acid substitutions in VOCs comparable to profiles identified by applying a fitness threshold to the combinatorial DMS library. Though diverse combinatorial sequences are generated by the simulations, open questions remain about how to translate these landscapes into epidemiological predictions. Collectively, these findings demonstrate the importance of training predictors on multi-mutation data and the capacity of ML to characterize higher-order mutations more accurately than inferring simple models from point mutations, but highlight difficulties with evaluating the performance of predictors across sequence space. The workflow presented here, implemented in Mavenets, involves training and evaluating bespoke machine learning models on DMS data to generate protein fitness landscapes. We anticipate that this framework is readily extensible to evolutionary prediction across a broad range of mutational data sets.

## Results and Discussion

### Machine Learning Architectures Predict RBD Binding Affinity

Prediction performance was first quantified by training and evaluating models on data extracted from the same DMS experiment. Multiple predictors were trained via least-squares regression to link RBD sequence to ACE2 binding affinity. The DMS data set used for training^6^ was experimentally initialized at the wild type (WT) RBD sequence and contains 105,525 variants with corresponding relative *K*_D_ values, reported as Δlog *K*_D_ relative to the WT, such that values above zero bind better than WT.

Predictors included linear models, a fully connected neural network, multilayer perceptron, (MLP) with one-hot embeddings and protT5 protein language model (PLM) embeddings,^16,53^ a message passing graph neural network (MPGNN),^54,55^ a transformer^53,56^ and gradient-boosted decision trees^57,58^ with and without dropout (DART);^59^ a highly performing subset of results are shown in (Figure 1). Among all searched models and hyperparameters (Tables S1–S3), a shallow MLP using a one-hot sequence embedding achieved the lowest mean absolute error (MAE) of 0.27 Δlog *K*_D_, outperforming linear, DART, T5 embeddings, transformer and a structure-based MPGNN with test MAEs of 0.93, 0.46, 0.34, 0.29, and 0.38 Δlog *K*_D_, respectively. Though more optimal hyperparameters and embeddings may exist, the strong performance of simple one-hot embeddings and MLP architecture on this data set are encouraging for conducting downstream tasks with these models with minimum overhead due to complex representation. These metrics were calculated using a randomly selected test set comprising 10% of the total data. A separate validation set of the same size was used for hyperparameter optimization, ensuring that the test set remained completely uninvolved during training. For the MLP with one-hot embeddings, two additional random training and validation splits with a fixed test set were tested, in addition to a completely new resplitting of the data, each showing similar performance (Figure S1).

**Figure 1:**
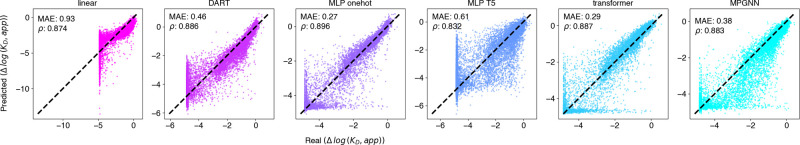
Summary of architectures’ performance on held out test set. Predicted versus experimentally measured Δlog *K*_D_ values are plotted with MAE and Spearman’s rank coefficients labeled on each plot.

An MPGNN with sufficiently expressive hyperparameters should ostensibly be able to outperform an MLP if defined via a suitable graph. The tested MPGNNs were created using nearest neighbor relationships found in the WT structure. Predicting the structure of protein variants is an outstanding challenge, as there is very little ground truth structural information for protein variants. Structure predictors often fail to predict the structural impact of individual mutations as they rely on solved structures and observed evolutionary data.^60,61^ Furthermore, many mutations prevent successful folding, presenting a challenge for structure prediction. Though design models have been shown to successfully identify stable mutations, they do so by identifying sequences that conform to the structural distributions of the Protein Data Bank (PDB), rather than explicitly predicting the structure of individual mutants.^62^ As demonstrated by Gelman et al.,^38^ a single wild type structure can be used for graph edge definition with high accuracy for many data points. However, similar to Wang and Gamazon,^44^ we did not see a performance gain from graph representation on this data set. Furthermore, since binding affinities are ensemble properties, static structural information may not be helpful as an inductive bias to predict mutational Δlog *K*_D_s in general.

The MLP with one-hot embeddings predicts the fitness of higher-order mutations with far greater accuracy than simply adding or averaging the impact of point mutations. Though unsurprising, this highlights the importance of accounting for nonadditive effects when analyzing DMS data, and the value of generating combinatorial mutation libraries.^12–15^ The MAE of the MLP remains low and relatively consistent regardless of the number of mutations, up to 9, the maximum number present in the training set (Figure 2A). Furthermore, we tested the impact of number of combinatorial mutations present during training on model performance and found a direct correlation (Figure S2). However, the number of data points decreases with higher mutation counts, and despite maintaining low MAE, the Spearman’s rank correlation coefficient drops significantly with more than six WT mutations, likely because these mutants tend to all fall in the low binding, high noise region (Figure 2B,C). This illustrates, as previously noted,^63,64^ that validating the accuracy of variant effect predictors is a complex task. Differences in intended use can change which aspects of estimated performance are relevant; therefore, consideration of multiple metrics is critical.

**Figure 2:**
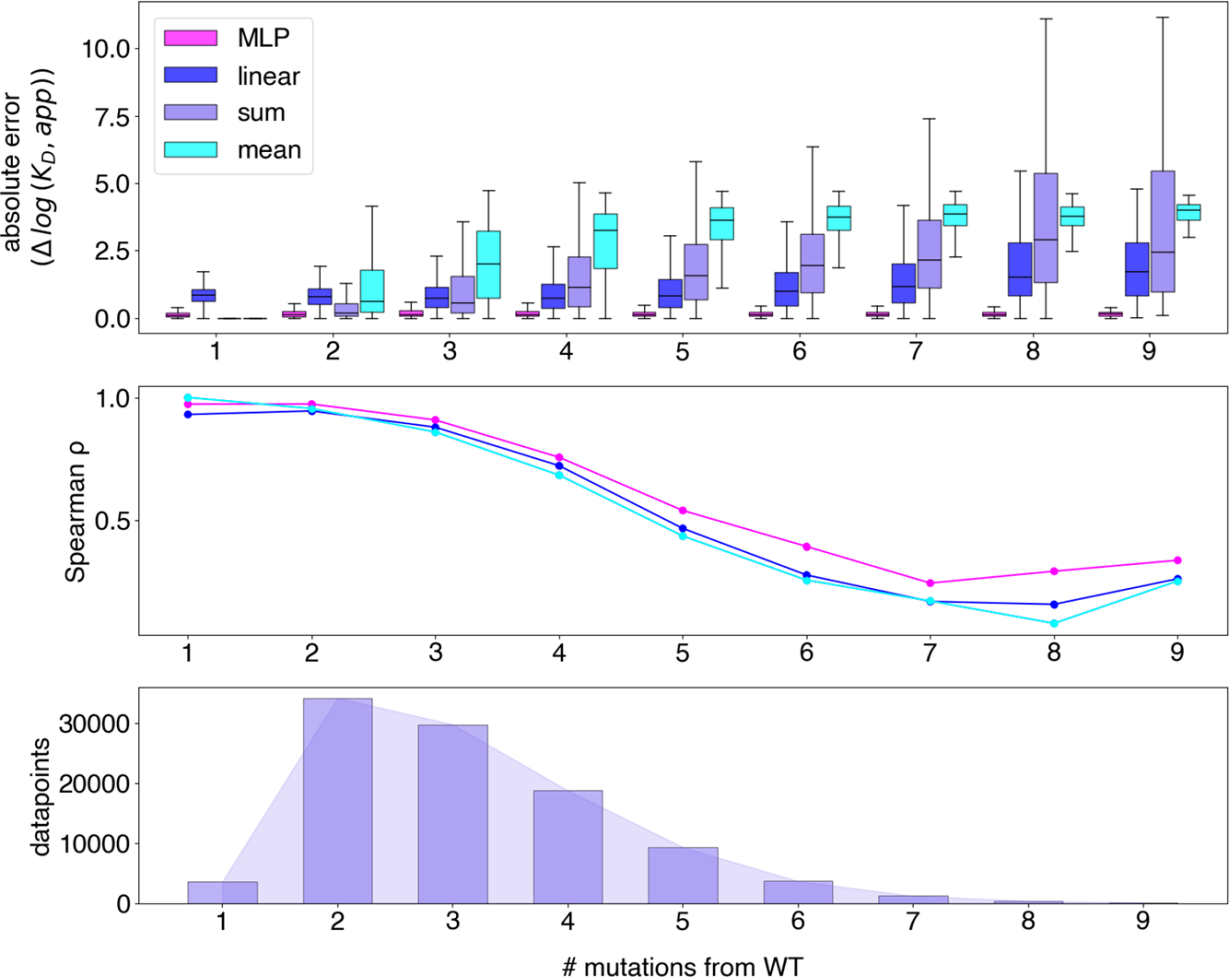
Predictions stratified by number of mutations from WT. (A) box and whisker plots showing absolute error between true and predicted Δlog *K*_D_ for sequences in the library predicted with the MLP with one-hot embedding, linear model, or by taking the sum or mean of each point mutation in the sequence. (B) Spearman’s rank correlation for each prediction in A. Note the sum and mean maintain the same rank and are therefore shown as a single cyan line. (C) Amount of sequences in library containing each number of mutations from WT.

#### Combining Multiple DMS Data Sets

Several VOC out-competed the WT strain early in the pandemic.^65^ As a result, subsequent RBD–ACE2 binding investigations were carried out with different starting sequences. In 2021 Bloom and colleagues published additional libraries initiated from N501Y, E484K, and B1.351 (N501Y, E484K, and K417N),^10^ which we will refer to as the beta study, followed in 2022 by libraries initiated from omicron BA.1 and omicron BA.2 data sets,^11^ which we refer to as the omicron study. These binding measurements followed the design of the original WT study; however new library construction techniques aimed at efficiently saturating point mutations decreased the data available by an order of magnitude (≈10^4^) per library. These new studies each included a control library constructed from the WT sequence, resulting in a total of seven new libraries in addition to the original study. Individual MLPs with one-hot embedding were trained on each library using the same method as above. Performance was evaluated on a random held out test set specific to each experiment. Models trained on these smaller data sets resulted in relatively low in-experiment performance with MAEs ranging 0.57 to 0.64 Δlog *K*_D_ compared to 0.27 Δlog *K*_D_ for models evaluated on the original data set; Spearman coefficients ranged from 0.730 to 0.825 compared to 0.896 in the original. These results are from individual hyperparameter scans to obtain the optimal network architecture for each data set (Figure 3A).

**Figure 3:**
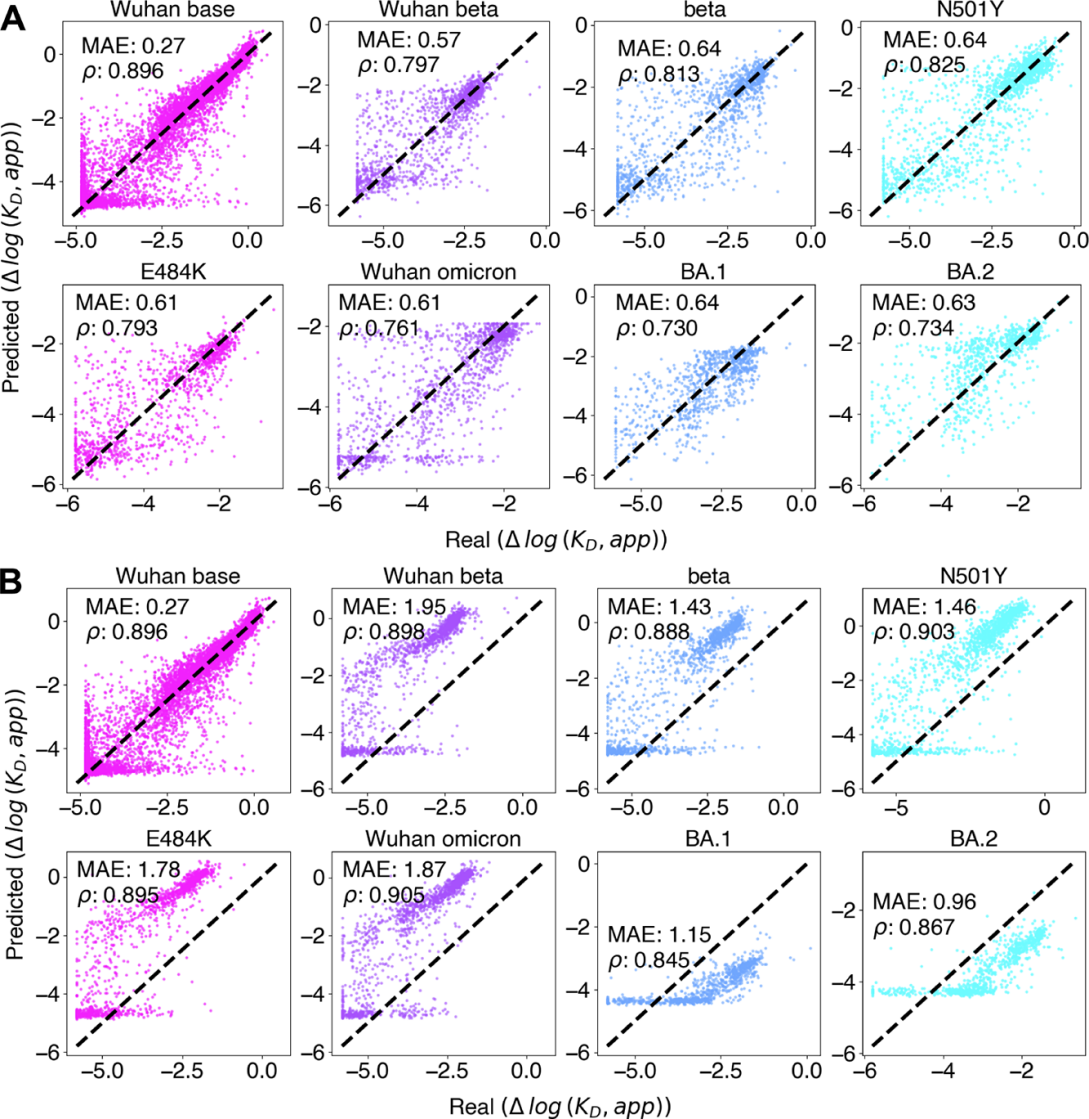
Summary of baseline extrapolation performance of initial model to 7 other DMS libraries. Each data set is labeled based on the starting sequence used to generate library. These were conducted in 3 separate studies by Starr et al., each with a Wuhan (WT) reference sequence control. Studies include the original (base),^6^ B.1.351 (Wuhan beta, beta, N501Y, E484K),^10^ and omicron (Wuhan omicron, BA.1, BA.2).^11^ (A) Predicted versus real Δlog *K*_D_ values on for independently trained models. (B) Predicted versus real Δlog *K*_D_ values of the original model on test sets from each individual library.

Since our model trained on the original WT library with one-hot embeddings showed relatively low MAE up to nine mutations away and high Spearman coefficients up to five mutations away from the WT sequence (Figure 2A,B), we sought to determine how well this model trained only on the original WT library could predict data generated from independent experiments. The beta experiment contains libraries with up to seven mutations from WT and the omicron experiment contains libraries with between 14 and 19 mutations from WT. Surprisingly, the Spearman correlation coefficients of predictions with the original WT-trained model were higher than those found by training on data from the same experiment for every library. We note this is true for one-hot embedding and that more optimal embeddings and hyperparameters may exist for these additional data sets. For example, BA.1 trained with T5 embeddings shows greater performance than one-hot with an MAE of 0.52 Δlog *K*_D_ and Spearman 0.828 (Figure S3). Nevertheless, we conclude the extrapolative performance of our model trained on the original data set is higher than anticipated, accurately ranking mutations from independent experiments, including those with greater combined mutation counts than in the training set.

To further investigate the extrapolative performance of the one-hot and T5 embeddings, we compared the prediction across data sets of models trained on the original data set or BA.1 data set with either set of embeddings (Figure S3). In the case of the original data set, the Spearman correlation coefficient was higher for models trained with one-hot embeddings, however for the models trained on only the BA.1 data set, the T5 embeddings showed superior extrapolative performance in four out of seven of the additional data sets. We hypothesize that the surprisingly high extrapolative performance of one-hot embeddings may be due to the nature of DMS data sets containing a single protein with high sequence identity to the WT, whereas the large corpus of protein sequences used to train PLMs could be less informative for distinguishing between these fine details. It remains to be determined if fine-tuning, alternative choices of PLMs, or modified data preparation strategies could further improve performance. The observed variability suggests that one should not draw conclusions on which architecture is optimal for all DMS data sets based on performance observations drawn from a single experiment. The combination of speed and accuracy of one-hot models is particularly attractive for downstream applications including Markov Chain Monte Carlo sampling, as described in the following section. Therefore, unless otherwise noted, the following experiments were carried out using models trained with one-hot embeddings.

Our conclusions regarding out of distribution performance are based on Spearman correlation coefficients, which indicate greater capacity to rank mutants rather than MAE. Absolute measurements, such as *K*_D_, are highly sensitive to assay conditions and significant variance exists between experiments. Therefore, when analyzing multiple DMS experiments, it is common to perform a data transformation to account for variance in experimental response curves,^25,40^ often making ranking a more robust metric of fitness. Visual inspection suggests that the large MAE are due to different experiment-specific offsets whereby the WT-trained model overpredicts affinity in all libraries besides omicron, where affinity is under-predicted (Figure 3B). A per-experiment linear calibration can reduce the MAE’s to a range of 0.26 to 0.56 Δlog *K*_D_ without impacting the ranking (Figure S4). This calibration, however, requires a priori knowledge of experimental offsets, which is not often available.

It is natural to ask whether the strong extrapolative performance of the WT-trained network on other smaller data sets can be improved by pooling training data from multiple experiments. First, fine-tuning the pretrained WT network with the data drawn from the smaller experiments was attempted; this did not significantly improve performance on low-data experiments. Instead, training MLPs from scratch (with one-hot embeddings) on combined data from all experiments proved more effective, resulting in a reduced MAE range of 0.38–0.46 Δlog *K*_D_ and increased Spearman correlation range to 0.893–0.907 for all data sets, except for those initiated from WT, which showed a drop in performance with 0.47–1.02 Δlog *K*_D_ MAE and 0.831–0.864 Spearman correlation (Figure 4A). The various libraries initiated from WT contain significant overlap in their sequences but contain different labels derived from each experimental setup. In order to account for the possibility of differing experiment response curves, a tuning head, which modifies the sequence-dependent prediction depending on the recorded experiment, was added to the MLP architecture.^66,67^ This decreased WT library MAEs to 0.28–0.41 Δlog *K*_D_, and increased the Spearman correlation range to 0.892–0.919. The non-WT libraries did not change substantially with this tuning (Figure 4B).

**Figure 4:**
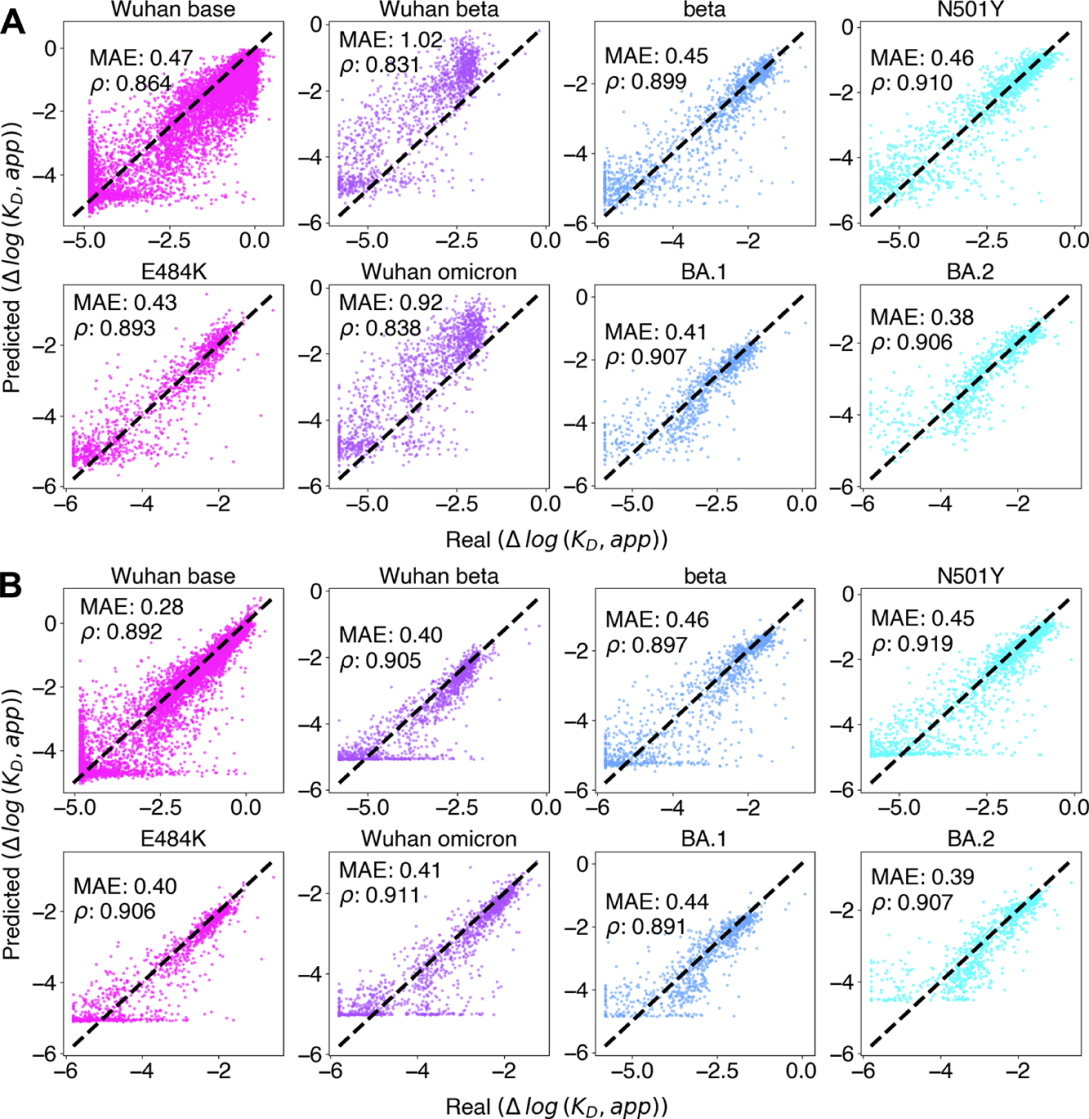
Summary of performance with aggregated training data from all DMS libraries. Each library is labeled based on the starting sequence as in Figure 3. Predicted versus real Δlog *K*_D_ values are plotted with MAE and Spearman’s rank coefficients shown for (A) without per-experiment tuning and (B) with per-experiment tuning.

We note that per-experiment tuning may be useful for certain analyses, but it is not straightforward to utilize this model for the goal of predicting the outcome of held-out experiments as the corresponding response curve is not known. As demonstrated by the model without per-experiment adjustments, an MLP alone has the capacity to capture the outcome of multiple experiments when those experiments characterize distinct locations in sequence space. Ideally, the predictive accuracy of these various models would be validated by comparing to a “true” binding affinity that is not subject to variable experimental response curves; however, the authors are not away of a test set with this property. Instead, accuracy is evaluated based on the capacity to predict epidemiological trends in viral fitness, as described in the next section.

### Characterizing the Fitness Landscape

To link our trained predictors to observed population mutation trends, trained MLPs with one-hot embedding that accurately predicted RBD binding affinity from sequence were used to explore estimated fitness landscapes underpinning future VOCs using MCMC.^68^ Multiple definitions were used to create the fitness surface from the prediction of binding affinity. First, simulations aimed to assess the ability to predict VOC mutation trends solely from information on the WT sequence, and therefore the original WT-trained predictor was used. Since no knowledge of VOCs was present in the original DMS used for training, this represents a rigorous assessment of extrapolative performance.

First, we propose that an informative landscape may be defined by assuming the system is characterized by a Boltzmann-like distribution

$$p(x)\propto f(x)=e^{-\beta \Delta \log K_{D}(x)}$$ 1

where *p*(*x*) is the probability of a particular sequence, *f*(*x*) is proportional to this probability, β is an unknown scalar, and Δlog *K*_D_ is the true change in binding affinity for a given mutant. In this formulation, Δlog *K*_D_ serves as a proxy for the relative change in binding free energy (ΔΔ*G*). By defining the landscape via a Boltzmann-like distribution, we treat higher-affinity variants (where Δlog *K*_D_ > 0) as occupying lower-energy, more probable states in the evolutionary landscape.

This definition suggests performing MCMC using the predicted output of a trained model in place of Δlog *K*_D_ with various values of β, with negative β concentrating sampling on more stable mutants. As MCMC allows the system to explore sequences that are highly dissimilar to those present in the training data, simulations of this type represent a complex evaluation of the extrapolative abilities of a fitness predictor. To sample from the distribution in eq 1, mutations were proposed by selecting a random position between 1 and 201 for random mutation to one of the 20 canonical amino acids. The proposed mutant was evaluated by the model and accepted or rejected according to the Metropolis criteria. Despite the promising out-of-experiment ranking performance, all simulations performed using eq 1 resulted in sequences with saturated mutagenesis (with every position containing a different amino acid compared to the WT) suggesting fundamentally limited model accuracy at high mutation levels (Figure S5). We also note that the combinatorial sequence space grows exponentially with sequence length; as a result, random mutation proposals are effectively biased toward highly mutated variants rather than few-site mutations.

Two modifications were made to produce more informative fitness simulations. First, to avoid excessive mutations driving sampling beyond mutation levels typical to the training data, sampled sequences were prohibited from exceeding 9 mutations relative to the WT sequence. However, the resulting simulations still accumulated mutations up to the set maximum, with very little sampling below 7 mutations (Figure S5), suggesting that errors in fitness predictions may exist for specific mutants within 9 mutations of the WT. Therefore, to produce physically plausible mutation statistics and further focus sampling, we further modified the sampled distribution to be proportional to eq 2

$$f^{\lambda }(x)=1[N(x)>\omega ]e^{-\beta g(x)-\lambda N(x)}$$ 2

where *g* corresponds to the predicted affinity, N is the number of residues which match those present in the WT sequence, λ controls bias toward the WT sequence, **1** denotes the 0–1 indicator function, and ω places a limit on the maximum number of mutations allowed during the simulation. λ may be viewed as a tuning parameter controlling the average number of mutations found during the simulation. Eq 2 is sampled by increasing the probability of proposing the WT amino acid at each position, while maintaining an equal probability of proposing non-WT amino acids, akin to the “favor native residue” function in Rosetta (Figure S5); this modified proposal mechanism controls the stationary distribution of the Markov chain (see Methods).

Eq 2 corresponds to a minimally adjusted form of e^–β*g*(*x*)^ modified to obtain a given average mutation count. Similar to eq 1, corrections of this form may be rigorously derived using maximum entropy arguments^69,70^ and are used to incorporate experimental information into molecular dynamics force-fields.^71–73^ While corrections are often applied to modify simulations to match known experimental observations, here we use this approach to flexibly penalize mutations without having a precise reference value for the target average mutation and fitness level; instead, β and λ were set to result in mutational fluctuations spanning 0 to 9 to increase overlap with the training set, and an average model prediction which overlaps with 0 while extending to higher values (Figure S5).

### Comparison of Simulations to Naturally Evolved Sequences

Sequences sampled from *f* ^λ^ were compared to the true sequence distribution observed in the human population deposited to Genbank as of Jan 31, 2025. These Genbank sequences represent total viral fitness, while the simulation distribution solely depends on predicted ACE2 binding affinity and sequence similarity to the wild type. Immune escape is a primary driver of SARS-CoV-2 evolution. As neutralizing antibodies compete with ACE2 for RBD binding, high fitness variants typically evolve to decrease antibody affinity while maintaining or increasing affinity for ACE2.^1–4^ Therefore, ACE2 binding affinity fitness offers a reasonable proxy for potential infectivity. However, this does not account for sequences which bind ACE2 strongly, but do not have high fitness due to maintaining or strengthening antibody binding. Furthermore, predicted mutations with high ACE2 affinity may negatively impact other stages of the viral life cycle, such as replication release of mature viral particles, which our models do not capture. Despite these scenarios leading to potential false positives regarding overall viral fitness, we sought to compare our ACE2 binding fitness landscapes with the sequences currently detected in the population.

Mutational frequencies present in the DMS data used for training were also compared against the Genbank data. As this DMS data contains random mutations that do not follow a fitness distribution dependent on ACE2 binding affinity, but rather reflect the experimental procedure used, samples were selected based on various Δlog *K*_D_ score cutoffs (Figure S6). An experimental Δlog *K*_D_ threshold of zero, corresponding to better or equal binding affinity compared to WT, was most predictive of VOC residue positions and mutations. Due to the large combinatorial sequence space, rather than focusing on exact sequence matches, we analyzed the propensity for mutation at each position. We also compared this to only analyzing greatest binding affinities from only the point mutation information, excluding combinatorial sequence information contained in the DMS data set.

To determine if an ML predictor trained solely on DMS data initiated from WT could predict mutations in VOCs, we sampled the distribution given by the baseline model. We found 25 M steps sufficient to achieve consistent mutation profiles in three independent MCMC replicates (Figure S7). We compared the top 20 mutations predicted by the simulations to those found in Genbank as of January 31st 2025. Over 70% of the simulation-predicted mutations occur at VOC residues, including positions 452, 493, 498, and 501. In contrast, just over 30% of the score threshold-identified mutations from the WT DMS data set overlap with VOC residues, including positions 339 and 452 (Figure 5A). The top scoring mutations out of the DMS point mutants only contained VOC positions 498, 501, and 505. Many residue positions are predicted to mutate to multiple amino acids. To account for this, we examined the top 20 residues with the highest mutation frequency, regardless of which amino acid they were mutated to, which revealed additional hotspot positions. The simulation additionally identified residue 373, while the DMS data highlighted positions 346, 493, and 501, and the point mutation only data identified 373, 477, and 484. In the top 5 positions, 4 out of 5 predicted by simulation overlap with VOC residues, compared to just 1 out of 5 from the DMS data and 2 out of 5 from the point mutations, although considering the top 20 positions results in the same total number of hotspots being predicted with each method (Figure S8). Collectively, this suggests that our simulations can enrich hotspot positions more effectively than analyzing the DMS data alone.

**Figure 5:**
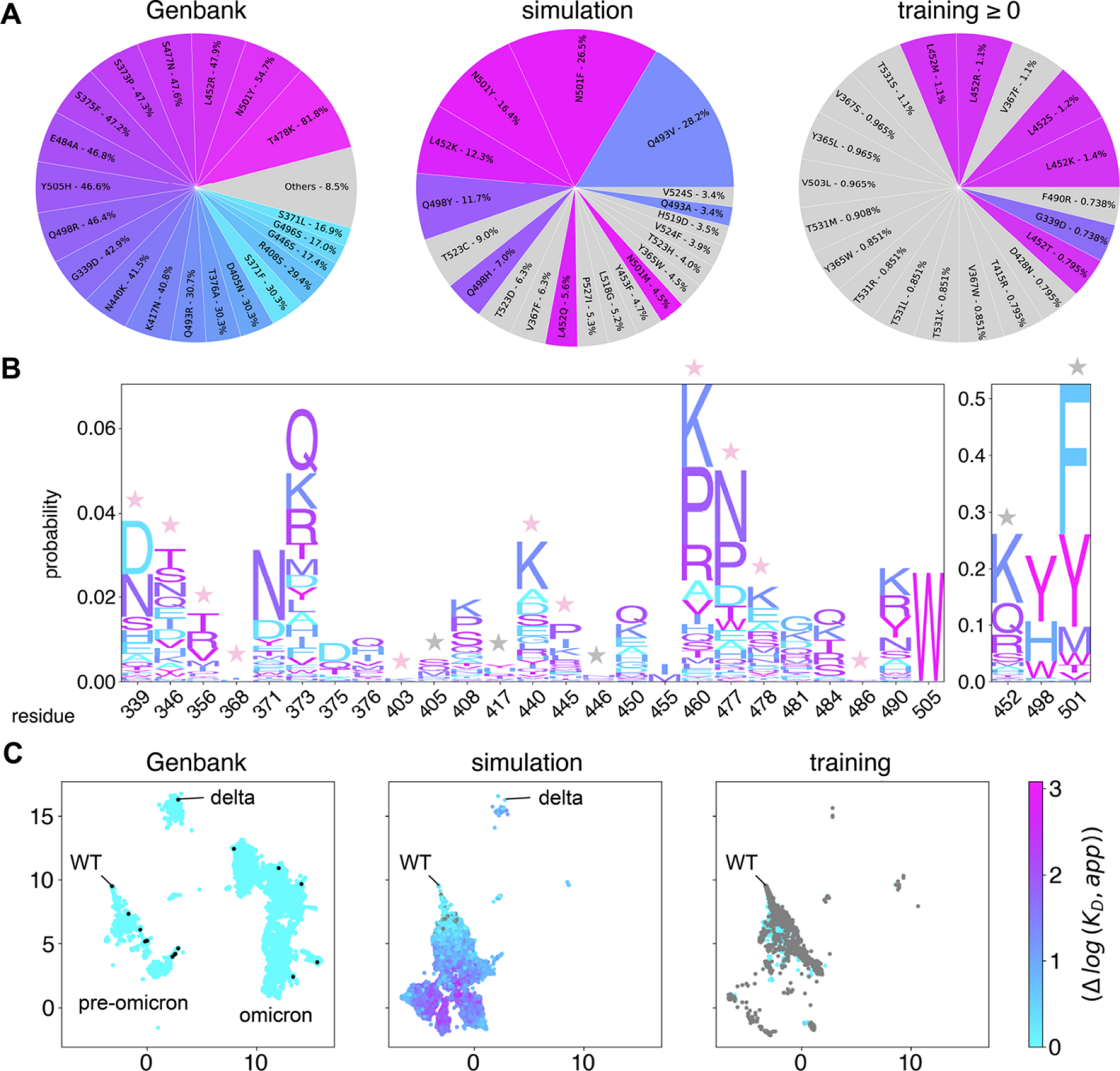
Sequence profile analysis. (A) Pie charts showing top 20 mutations found in sequences from Genbank, simulation from WT with MLP trained on original WT data, and the training data sequences with scores greater than or equal to 0 Δlog *K*_D_ (better than WT). The percentage corresponds to the amount of total sequences in a given set have that mutation. Wedges are colored based on residue number, corresponding to those in the top 20 Genbank mutations. (B) Logo plot of mutations in sequences from simulation for the 28 VOC residues. A pink star indicates the most probable amino acid at a given position corresponds to a VOC mutation. A gray star indicates the most probable amino acid has the same physiochemical properties as the VOC mutation, such as polar, basic, and aromatic. (C) UMAP of one-hot encoded sequences shown in A colored by Δlog *K*_D_. Unique Genbank sequences were colored with a score of zero, scores less than zero (i.e., worse binders than WT) are colored gray. VOC from (Table 1) are shown as black dots on the first plot with representative variant categories labeled.

Next, the most frequent substitution at each position was analyzed for 28 mutations found in VOC strains (Table 1). Our simulation always predicted the VOC amino acid as the most frequent mutation at nine positions: G339D, R346T, R403K, N440K, V445P, N460K, S477N, T478K, and F486P, while K356T and L368I were predicted as the most frequent in some replicates, but the second most frequent in others (Figures 5B and S7). All of the correctly predicted mutations aside from S477N and T478K are specific to omicron strains. Additionally, at several other positions, the simulation did not predict the mutation found in VOC as the most frequent, however it predicted an amino acid with similar physiochemical properties. For example, N501 was often predicted to mutate to F instead of Y and L452 to K rather than R. Comparing this to the DMS or point mutation data, nine mutations were also predicted, however their identities differed. Mutations R346T, S477N, and F486P were predicted by the simulation but not DMS thresholding, and N481K, and N501Y were identified with DMS thresholding but not the simulation. Analysis of the highest scoring point mutants identified F486P, which was found in the simulation but not thresholding, but did not identify T478K which was found in both. Similar to the simulation, point mutation at position N501 to F has a higher affinity than to Y. Though the MCMC parameters β, *C*, and ω were initially chosen without inputting knowledge of the VOC, we analyzed their influence on VOC hotspot residue and amino acid substitution prediction. No parameters were found to improve predictions (Table S4). This analysis suggests comparable performance for predicting the most frequent substitution to VOC residues with the simulation and analysis of DMS data alone, though some mutations were only predicted by the simulation, while others were identified with the DMS but not the simulation.

**Table 1:** Summary of Variants of Concern^a^

name	strain	RBD mutations
Alpha	B.1.1.7	E484K, **N501**Y
Beta	B.1.351	**K417**N, E484K, **N501**Y
Gamma	P1	**K417**T, E484K, **N501**Y
Delta	B.1.617.2	**L452**R, **T478K**
Epsilon	B.1.42(7/9)	**L452**R
Eta	B.1.525	E484K
Iota	B.1.526	E484K
Iota’	B.1.526	**S477N**
Kappa	B.1.617.1	**L452**R, E484Q
Zeta	P2	E484K
Mu	B.1.621(0.1)	**R346**K, E484K, **N501**Y
21K Omicron	BA.1	**G339D**, S371L, S373P, S375F, **K417**N, **N440K**, **G446**S, **S477N**, **T478K**, E484A, **Q493**R, G496S, **Q498**R, **N501**Y, **Y505**H
21L Omicron	BA.2	**G339D**, S371F, S373P, S375F, T376A, **D405**N, R408S, **K417**N, **N440 K**, **S477N**, **T478 K**, E484A, **Q493**R, **Q498**R, **N501**Y, **Y505**H
22A/B Omicron	BA.4/5	**G339D**, S371F, S373P, S375F, T376A, **D405**N, R408S, **K417**N, **N440 K**, **L452**R, **S477N**, **T478 K**, E484A, **F486**V, **Q498**R, **N501**Y, **Y505**H
23A Omicron	XBB.1.5	**G339**H, **R346T**, **L368I**, S371F, S373P, S375F, T376A, **D405**N, R408S, **K417**N, **N440 K**, **V445P**, **G446**S, **N460 K**, **S477N**, **T478 K**, E484A, **F486P**, F490S, **R493**Q, **Q498**R, **N501**Y, **Y505**H
24A Omicron	JN.1	**G339**H, **K356T**, S371F, S373P, S375F, T376A, **R403K**, **D405**N, R408S, **K417**N, **N440 K**, **V445**H, **G446**S, N450D, **L452**R, L455S, **N460K**, **S477N**, **T478K**, N481K, V483-, E484K, **F486P**, **Q498**R, **N501**Y, **Y505**H

a Mutations predicted by the simulation are shown in bold. Omicron variants names are also labeled with Nextstrain clade scheme.^80^

Though the analysis here is focused on sequence-based prediction, it is important to note that the biological effects of different RBD sequences are driven by physical differences in the interactions between RBD and ACE2. The structural, energetic, and physicochemical factors underlying VOC have been extensively studied elsewhere.^74–79^ However, we highlight that there are substantial advantages of a sequence-based workflow for predicting VOC using only knowledge of the WT sequence (e.g., when studying emerging viruses for which structures are not yet known).

As the models in this article were were trained to minimize error on held out data, their predictions may remove noise intrinsic to DMS measurements. This raises the question: can replacing experimental observations with predictions improve thresholding on DMS data sets? To assess whether this could improve prediction of VOC mutations, we performed inference on the training set and repeated analysis using the resulting Δlog *K*_D_ values for score thresholding. This resulted in a similar hotspot prediction to the original thresholding (Figure S9), indicating the simulation still performs better hotspot prediction. However, when considering the most frequent amino acid at the select VOC positions, the “denoised” score threshold resulted in a sequence profile with 13 of the most frequent substitutions correctly overlapped with the VOC mutations, including those identified by the simulation and experimental score threshold except for G339D and T478K. Additional mutations S371L, R408S, G446S, Q498R were identified with the denoised score threshold, but not by the simulation, experimental score threshold, or point mutation analysis (Figure S10). Though the simulation performed similarly to the DMS data with a threshold, the ML model-denoised thresholding of DMS data performed best at the task of retrospectively predicting the most probable mutation at a given position.

Simulations were next initiated from WT using the model trained on all DMS libraries using per-experiment tuning. Since these libraries contain information on beta and omicron BA.1 and BA.2 strains, predictions were assessed by focusing on 12 of the 28 mutations which appeared after BA.2 including BA.4/5, XBB.1.5 and JN.1 (Table 1). However, neither simulation of the multiexperiment model or thresholding of the corresponding DMS data identified new hotspots or substitutions not present in simulations performed by the original model. Both the original and aggregated training data identified the post-BA.2 residue 452 as most frequently mutated, while the original data also identified residue 490. Considering positions rather than frequent mutations, both additionally identify residue 346. Residues 452 and 460 are in the top 20 of both simulations trained on either model, with 460 representing another hotspot predicted by the simulations but not the DMS alone (Figure S11). The most frequent substitution at each position was the same as the original WT-trained model for the aggregate training data and nearly the same for the simulation, however the simulation did not predict R346T. The denoising was not as helpful with this model either, no additional substitutions were identified and L368I, V445P, N481K, F486P, where not correctly predicted, while L452 was predicted as K instead of R (Figure S12). This suggests that inclusion of additional DMS libraries did not improve prediction of the most frequent substitution, and worsened hotspot prediction. This may be attributed to difference in library construction techniques leading to smaller distributions around the starting sequence, or differences in experimental response offsets; however, the exact cause is not clear. Centering the simulation bias around the BA.1 sequence did not result in additional hotspot residue or VOC mutation predictions either. Simulations with either model were less predictive than the simulations initiated from the WT sequence with the model trained on the original data set (Figure S13).

The combination of strong prediction accuracy on DMS data sets combined with the tendency of unbiased simulations to quickly accumulate saturating mutagenesis suggests while these predictors contain substantial information about parts of sequence space, certain regions uncharacterized in the training data create substantial challenges for simulation. While the introduction of additional DMS training data sets increases this coverage, they did not result in an increased prediction of VOC. This increase in data is dwarfed by the combinatorial growth in sequence space; for example, the omicron data set provides 10^4^ additional data points; however, there exist more than 10^18^ possible mutants within its characteristic edit distance to the WT. Furthermore, as demonstrated by DMS denoising using a predictor trained with multiple data sets, improved performance on MAE across data sets may not translate to better downstream predictions of relevant mutations. Despite these challenges, simulations which generate informative mutations can be produced by biasing sampling to higher confidence areas of sequence space.

In order to better understand the overlap between the simulations, training data sets, and experimentally observed sequences we used principal component analysis (PCA) followed by Uniform Manifold Approximation and Projection (UMAP)^81^ to project one-hot encoded sequences in two dimensions. When dimensional reduction was trained using only the simulated and VOC sequences, clusters showed overlap with all VOCs (Figure S14) using the model trained only on the WT data. However, when dimensional reduction was repeated using a combined set of sequences from Genbank, DMS training set, and the simulation, VOCs clearly clustered into three distinct regions including preomicron, delta, and omicron variants in the Genbank sequences. The simulated sequences primarily appear to explore preomicron and delta sequences, in addition to new unexplored regions. The DMS score thresholded sequences only appear to represent the preomicron sequences (Figure 5C). The dependence of the apparent coverage of VOCs on the reference set used for dimensional reduction underscores the sensitivity of visualization methods such as UMAP to input data selection. Despite this variability, however, differences in coverage between the data used for training and that produced by simulation underscore the extent to which extrapolation drives simulation results. The lack of overlap between simulation and omicron sequences is not surprising due to the biases required to drive simulation accuracy. Accurately characterizing sequence landscapes remains an open area of research with a range of strategies available,^82–84^ representing a promising avenue for future investigation.

## Conclusions

We have demonstrated that a combination of supervised ML with DMS and MCMC is a viable approach for simulating fitness landscapes and extracting meaningful information on possible mutations. Using a multilayer perceptron, we achieved highly accurate predictions of the SARS-CoV-2 RBD binding to ACE2. Notably, these predictions outperformed additive or averaged estimates in capturing the effects of combinatorial mutations suggesting an understanding of epsistatic relationships. Through retrospective comparison of predictions to population data, this predictor was shown to approximate an informative fitness landscape when combined with a maximum-entropy bias, generating a large number of unique combinatorial RBD sequences, identifying hotspot residues, and predicting mutations present in VOC (including omicron clades) from models only trained on variants near the WT sequence. Though unique VOC mutations were identified, aggregate sequence profiles did not identify a greater number of VOC mutations compared to analysis of high fitness sequences in the DMS data alone, leaving an open question of how to best translate generation of sequences with high predicted fitness into concrete epidemiological predictions. One limitation of the current implementation of evolutionary simulations is the reduction of viral fitness to the single metric of ACE2 binding. Furthermore, since our simulations derive from DMS experiments conducted in a controlled laboratory, they inherently do not account for additional biological variables such as population size, selective pressure scaling, or environmental stability, representing avenues for future improvement. We introduce the open source package Mavenets: https://github.com/SztainLab/mavenets for reproducing and extending these findings, laying the groundwork for development of improved evolutionary predictors which may include multiple fitness metrics, alternative sampling algorithms, and prospective variant testing. Though only applied here to the case of SARS-CoV-2, these methods have potential application to a broad range of MAVE experiments and the in silico evolution of viral, bacterial, cancer-related, and designed proteins.

## Methods

### Data Preparation

The WT ACE2–RBD binding DMS data set was obtained from https://media.githubusercontent.com/media/jbloomlab/SARS-CoV-2-RBD_DMS/master/results/binding_Kds/binding_Kds.csv. Additional details on the data set generation can be found in the SI. We filtered the original base data set containing 195,081 barcoded sequences from two replicate libraries. We removed those with NaN values in the “delta_log10Ka” column, resulting in 146,437 sequences. Note that “delta_log10Ka” refers to Δlog *K*_D,apparent_, not to be confused with the association constant, *K*_A_. The “apparent” nomenclature was adopted to account for the fact that dimeric ACE2 was used in the study.^6^ There were 11,672 duplicate sequences within each of the libraries. Given the low standard deviation of Δlog *K*_D_ values, we took the mean of these duplicates. This resulted in a total of 108,588 sequences. Next, 3,063 duplicates across the two libraries remained, with a similarly low standard deviation. Averaging these resulted in the final 105,525 unique sequences with Δlog *K*_D_ values used in this study. Additional data obtained from experiments conducted by Bloom and colleagues for the beta^10^ and omicron^11^ variants were constructed by purchasing genes for site saturation mutagenesis. Synthesis and PCR errors inevitably result in sequences with multiple mutations, resulting in library sizes around 10^4^. All universal resource locators for additional data sets are provided in Table S5. Each of these were filtered and processed in the same manner as the original, however the Δlog *K*_D_ was obtained by subtracting the WT log *K*_D_ as measured in the original library.

Data was randomly split into 80/10/10% points for train, valid, and test, respectively; except for training of DART models where 5-fold cross validation was performed over the combination of these train and validation sets.

### Prediction Models

#### Multilayer Perceptron

A multilayer perceptron (MLP) implemented with PyTorch^85^ was trained on various sequence embeddings to predict Δlog *K*_D_. Networks were created with ReLU^86^ activation on all layers except the output layer, which used no activation function. The mean squared error (MSE) between predicted and ground truth Δlog *K*_D_ values was used as the loss function for training. A batch size of 32 with the AdamW optimizer^87,88^ was used to train until the convergence was observed via an uptick in the validation loss or a maximum number of training epochs were reached; additional details on training are found in the SI. No learning schedule was used with the optimizer; however, the schedule free optimizer^89^ was attempted during early hyperparameter tuning but did not produce better results. MLPs trained on one-hot encodings were characterized by considering hidden layers of the following sizes: 8, 16, 32, 64, 128, and 256. In the case of MLPs trained on T5 embeddings, a secondary set of networks with hidden layer sizes of 128, 256, 512, and 1024 were additionally considered; while beneficial to T5-based MLPs, these larger layers did not improve one-hot based MLPs. Networks with hidden depths of 0–3 were created by independently varying the size of each layer (a depth of 0 corresponds to the linear model). In addition, scanning was performed over learning rates 3 × 10^–4^ and 1 × 10^–4^ and weight decay was scanned over 5 × 10^–3^, 1 × 10^–3^, 5 × 10^–4^, and 1 × 10^–4^, with larger networks additionally scanning over dropout levels 0, 0.1, 0.3, and 0.5.

These various architectural and hyperparameters were explored via a grid search (Tables S1 and S2) We note that many MLP hyperparameter choices performed close to the optimal selected architectures; as a result, we do not attempt to interpret the optimal hyperparameters found for any experiment. Once optimal architectural and hyperparameter details were determined, these settings were used to train 3 networks with differing random initializations; the best performing model instance on the validation set was then evaluated on the test set.

##### Sequence Embedding

Multiple different sequence embeddings (i.e., featurizations) were used as input to the MLP during early architecture explorations. A one-hot embedding was utilized based on the 20 canonical amino acids. Alternatively, a glycan one-hot embedding was defined by adding an additional token to the one-hot embedding denoting glycosylated asparagine residues based on the Nx­[S/T] sequence motif.^90^ A UniRep^91^ embedding was used with the TAPEtokenizer^92^ utilizing a 1900-dimensional hidden state layer. SeqVec embeddings generated via Bio Embeddings were additionally tested,^93^ as were ProtTrans-Bert-BFD, ProtT5-XL-UniRef50,^53^ and ESM1b^94^ embeddings with default settings. The hyperparameters considered are outlined in Table S2. Additional details on input representations, including those used for the MPN an transformer, are found in the SI.

Other modeling approaches used only the one-hot sequence embedding for testing, with the exception of DART models which utilized equivalent categorical variables based on residue type and the transformer which used a custom scheme for embeddings.

#### Message Passing Graph Neural Network

A message passing graph neural network was created using PyG^95,96^ to operate on a graph defined by a single structure of the WT RBD obtained from simulations performed by Barros et al.^97^ Unless otherwise noted, training details mirrored those used for the MLPs. The structure was extracted from the trajectory by first clustering using cpptraj^98^ via the hierarchical method with a random sieve of 10; this produced a total of 20 clusters. The average structure from the most populated cluster was then used to generate a residue graph with mdtraj.^99^ A one-hot residue representation of each sequence was used to describe the nodes, while edges were defined based on the distance between alpha carbons of each residue in the structure. In the optimal architecture, edges were connected based on a window in sequence space of 10 residues in the N and C terminal directions, as well as a distance cutoff of 15 Å in 3D space. Distances were featurized into 10 dimensional feature vectors using a Gaussian expansion and scaled with a factor of 75. Messages were passed 7 times, each containing an MLP with a 128 feature hidden layer. The following hyperparameters were scanned to identify the optimal architecture: window sizes: 5, 10, 15, and 20 Å; distance cut off: 10, 15, 20, 30 Å; distance feature vector dimensions: 0, 6, 8, 10, 12; scaling factor: 1, 10, 25, 50, 75, 100.

#### Transformer

A BERT-style^100^ (encoder-only) transformer was implemented in Pytorch using the SiLU activation function.^101–103^ Unless otherwise noted, training details mirrored those used for the MLPs. The learning rate was set to 3 × 10^–4^. Embeddings were initialized by summing an embedding drawn from a look-up table based on residue type with an embedding vector specific to each position in the sequence. These embeddings are passed through multihead attention, layer norms, and an MLP as described in the pre-LN architecture.^104^ After Multi-head attention-based updates, each of the resulting embeddings is passed through a single shared MLP to produce a scalar value; this value is summed over the entire sequence to produce the model prediction. Hyperparameter optimization was performed scanning over the number of attention blocks, the number of attention heads, the embedding dimensionality, the dropout rate of the block MLPs, the dropout rate of the attention modules, the number of layers in the final MLP, and the dropout rate of the final MLP. A table of scanned values is found in Table S3.

#### Multiple Additive Regression Trees with Dropout

Multiple Additive Regression Trees with Dropout (DART)^59^ and gradient boosted decision tree models^57,58^ were created using the Light Gradient Boosted Machine library.^105^ For brevity, both gradient boosted decision trees (without dropout) and Multiple Additive Regression Trees with Dropout are referred to as DART. The optimal model was found to use 511 leaves, dropout, 1399 trees, a drop rate of 0.3, a maximum drop of 50, minimum leaf data size of 1, a categorical l2 of 0, and a categorical smoothing of 5. Hyperparameters were scanned over using multiple local grid searches, scanning over the presence of dropout (i.e., DART or gradient boosted decision trees), number of leaves, the rate of tree dropping, the presence of categorical smoothing and l2, the rate of tree dropping, and the minimum number of data allowed per leaf.

All hyperparameter combinations were trained for 5000 updates to determine the optimal number of trees. We note that a relatively large number of leaves was found to be beneficial to regression despite the low number of training points, likely implying a high degree of epistasis. Unlike other methods, hyperparameters were optimized using 5 fold cross validation performed on the combined train and validation data sets used for other methods. All attempts treated inputs as categorical variables.

##### Tuning Heads

Certain neural networks were trained with per-experiment tuning (Figure 4B). This was implemented by first transforming the sequence into a scalar value using an internal network and subsequently refining this scalar value using function parametrized by the experiment identity; this is described in eq 3, where *g* denotes the full predictive model, *h* refers to an internal network, and *m* denotes the experimental adjustment.

$$g(x_{\mathrm{seq}},x_{\exp})=m(h(x_{\mathrm{seq}}),x_{\exp})$$ 3

*x*_exp_ was defined to be an integer unique to each experiment and *x*_seq_ corresponds to the protein sequence. The internal network was set to be an MLP for the results shown in Figure 4B; results utilizing the transformer architecture yielded similar results (not shown). The experimental adjustment was defined to be a MLP with one hidden layer (input and output dimension of one) that is coupled to the output of *h* via a residual connection; ELU^106^ was used as the activation function to avoid dying neurons. A residual connection was chosen after nonresidual tuners resulted in unreliable training convergence, impeding hyperparameter searches during early architecture experiments. ELUs were chosen to avoid abrupt changes in per-experiment response curves. The final (i.e., output) layer of this MLP is specific to a single experiment and other parameters are fully shared across experiments. In all cases hyperparameters of the per-experiment tuning head were optimized alongside those of the internal network. When training models using tuning heads, the first 20 training epochs used a linear combination of losses: the first loss corresponded to the mean squared error of the internal network and the second loss corresponded to the mean squared error of the experiment-adjusted prediction. Training was started weighting these two losses equally and linearly progressed to only penalizing the experiment-adjusted prediction by epoch 20; all remaining training was performed using only the experiment-tuned output. This scheduling was observed to stabilize training. The scanned sizes of the hidden layer were 1, 2, 4, 8, 16, 32, 64, 128, and 256.

### Comparing Combinatorial Predictions

To compare higher-order mutation prediction with MLP versus adding or averaging the impact of point mutations, values of Δlog *K*_D_ for point mutations were obtained from the original WT data set. This library contained 3602 unique point mutation values, corresponding to 89.6% coverage of saturating point mutations. Sequences in the library containing substitutions without a corresponding point mutation were removed from analysis. Sequences were stratified based on the number of mutations, and evaluated either with the MLP, the linear model, or by adding or averaging the Δlog *K*_D_ of each mutation present. The “linear” model, as shown in Figure 1, was trained as a MLP with no hidden layer on a one-hot encoding.

### Monte Carlo Simulations

Markov Chain Monte Carlo (MCMC) simulations were performed to explore fitness landscapes implied by the output of trained models. For MCMC simulation a random position between 1 and 201 was selected for mutation to one of 20 canonical amino acids. The proposed mutant was evaluated by the model and accepted or rejected according to the Metropolis criteria.

MCMC performed using our trained model in place of Δlog *K*_D_ in eq 1 with Metropolis-Hasting^107^ moves leads to problematically high levels of mutations, likely driven by limited prediction accuracy on mutants far from those present in training set (SI). MCMC simulations are instead performed to target the following native-biased probability function

$$p^{\lambda }(x)\propto f^{\lambda }(x)=1[N(x)>\omega ]e^{-\beta g(x)-\lambda N(x)}$$ 4

where *x* denotes a amino acid sequence, N denotes the number of residues in *x* which match those present in the WT sequence, λ ∈ (−*∞*, *∞*) determines a bias toward the WT sequence, **1** denotes the 0–1 indicator function, and ω places a limit on the maximum number of mutations allowed during the simulation. ω was set such that mutants with greater than 9 mutations were not considered. Several values for β were scanned including −5, −10, −15, −20, and −10 was chosen to keep the distribution of Δlog *K*_D_ outputs overlapping with and above 0, equal to or greater than the fitness value of the WT.

Eq 4 is targeted by proposing single-residue mutations and accepting sequences using a modified form of the Metropolis procedure. Candidate 1-mutations were proposed using

$$q\left(x^{\prime }|x\right)=q\left(n,r|x\right)\{\begin{array}{ll} \frac{C}{L}, & r={\mathrm{WT}}_{n}\\ \frac{1-C}{19L}, & r\neq {\mathrm{WT}}_{n} \end{array}$$ 5

where *n* determines the index of the residue to mutate, *r* determines which amino acid the mutation will result in, and *L* denotes the number of mutable residues. Note that this a joint probability mass function over *n* and *r*; however, *n* does not appear as the corresponding marginal is uniform. Furthermore, note that this proposal distribution is asymmetric.

Proposed mutations are accepted according to a Metropolis criterion given by the following probability:

$$\alpha \left(x^{\prime }|x\right)=\min\left[1,\frac{e^{-\beta g\left(x\prime \right)}}{e^{-\beta g\left(x\right)}}\right]$$ 6

Application of eqs 5 and 6 result in the stationary distribution given by eq 4 when defining λ to be $-\ln\frac{19C}{1-C}$ due to proposal asymmetry. Values for *C* were also tested and 0.95 was chosen to balance the diversity of the mutation landscape (Figure S5). The selected value for *C* corresponds to λ = 5.9. The final acceptance is performed by drawing a variate from a uniform distribution between 0 and 1 and comparing it to eq 6. If accepted, the mutant is set to be the current state of the MCMC simulation; if rejected, the state of the simulation is not changed. MCMC simulations with 25 million steps were observed to return equivalent results on repeated runs, implying that simulations are well-converged.

### Genbank Sequence Collection

A total of 9,017,742 complete SARS-CoV-2 Receptor Binding Domain genome sequences were downloaded from NCBI Genbank^108^ on Jan 31, 2025 by filtering for the SARS-CoV-2 taxon (taxid:2697049), human host, and a minimum length of 29,000 nucleotides. The 603bp RBD region (603 bp) from the Wuhan-Hu-1 reference genome (accession NC_045512.2,^109^ positions 22552–23155, NCBI RefSeq) served as the alignment target.

#### Sequence Alignment and Filtering

The pipeline used multiprocessing to parallelize alignment of the 257 GB data set and sequences were loaded to a bounded queue to manage memory constraints. Alignments were performed with BioPython’s^110^ PairwiseAligner with parameters mismatch score: −1 and gap opening penalty: −2. A windowed alignment strategy was employed during alignment focusing first on the expected RBD region with iterative expansion before falling back to full sequence alignment when necessary. Ambiguous nucleotides (N) replaced with their corresponding Wuhan reference nucleotides. The 603-nucleotide RBD coding sequences were converted to 201 amino acids using the standard genetic code table. Sequences exhibiting >20% divergence from reference in the RBD region were excluded (<500 sequences). The final data set after alignment and filtering yielded 8,255,834 RBD sequences (91.55% of total), representing 27,980 unique RBD sequence variants consisting of combinations of 1311 distinct mutations.

## Supplementary Material



## Data Availability

All code necessary for featurizing, training, and evaluating models and for running MCMC simulations can be found at: https://github.com/SztainLab/mavenets. Code for reproducing all figures in the manuscript can be found at https://github.com/SztainLab/ML_evolution_SARS-CoV-2. Links to DMS data used can be found in (Table S5).

## References

[1] Harvey W. T., Carabelli A. M., Jackson B., Gupta R. K., Thomson E. C., Harrison E. M., Ludden C., Reeve R., Rambaut A., Peacock S. J. (2021). SARS-CoV-2 variants, spike mutations and immune
escape. Nature Reviews Microbiology.

[2] Krause P. R., Fleming T. R., Longini I. M., Peto R., Briand S., Heymann D. L., Beral V., Snape M. D., Rees H., Ropero A.-M. (2021). SARS-CoV-2
variants and vaccines. New England Journal of
Medicine.

[3] Miller J., Hachmann N. P., Collier A.-r. Y., Lasrado N., Mazurek C. R., Patio R. C., Powers O., Surve N., Theiler J., Korber B. (2023). Substantial neutralization escape by SARS-CoV-2 Omicron
variants BQ. 1.1 and XBB. 1. New England Journal
of Medicine.

[4] Markov P. V., Ghafari M., Beer M., Lythgoe K., Simmonds P., Stilianakis N. I., Katzourakis A. (2023). The evolution of SARS-CoV-2. Nature Reviews Microbiology.

[5] Piccoli L., Park Y.-J., Tortorici M. A., Czudnochowski N., Walls A. C., Beltramello M., Silacci-Fregni C., Pinto D., Rosen L. E., Bowen J. E. (2020). Mapping
neutralizing and immunodominant sites on the SARS-CoV-2 spike receptor-binding
domain by structure-guided high-resolution serology. Cell.

[6] Starr T. N., Greaney A. J., Hilton S. K., Ellis D., Crawford K. H., Dingens A. S., Navarro M. J., Bowen J. E., Tortorici M. A., Walls A. C. (2020). Deep mutational scanning of SARS-CoV-2 receptor
binding domain reveals constraints on folding and ACE2 binding. cell.

[7] Hoffmann M., Kleine-Weber H., Schroeder S., Krüger N., Herrler T., Erichsen S., Schiergens T. S., Herrler G., Wu N.-H., Nitsche A. (2020). SARS-CoV-2
cell entry depends on ACE2 and TMPRSS2 and is blocked by a clinically
proven protease inhibitor. cell.

[8] Beyerstedt S., Casaro E. B., Rangel É. B. (2021). COVID-19: angiotensin-converting
enzyme 2 (ACE2) expression and tissue susceptibility to SARS-CoV-2
infection. European journal of clinical microbiology
& infectious diseases.

[9] Jackson C. B., Farzan M., Chen B., Choe H. (2022). Mechanisms
of SARS-CoV-2
entry into cells. Nat. Rev. Mol. Cell Biol..

[10] Starr T. N., Greaney A. J., Hannon W. W., Loes A. N., Hauser K., Dillen J. R., Ferri E., Farrell A. G., Dadonaite B., McCallum M. (2022). Shifting
mutational constraints in the SARS-CoV-2
receptor-binding domain during viral evolution. Science.

[11] Starr T. N., Greaney A. J., Stewart C. M., Walls A. C., Hannon W. W., Veesler D., Bloom J. D. (2022). Deep mutational scans for ACE2 binding,
RBD expression, and antibody escape in the SARS-CoV-2 Omicron BA.
1 and BA. 2 receptor-binding domains. PLoS pathogens.

[12] Starr T. N., Thornton J. W. (2016). Epistasis in protein
evolution. Protein science.

[13] Yang K. K., Wu Z., Arnold F. H. (2019). Machine-learning-guided
directed evolution for protein
engineering. Nat. Methods.

[14] Freschlin C. R., Fahlberg S. A., Romero P. A. (2022). Machine
learning to navigate fitness
landscapes for protein engineering. Curr. Opin.
Biotechnol..

[15] Taft J. M., Weber C. R., Gao B., Ehling R. A., Han J., Frei L., Metcalfe S. W., Overath M. D., Yermanos A., Kelton W. (2022). Deep mutational learning predicts ACE2 binding and
antibody escape to combinatorial mutations in the SARS-CoV-2 receptor-binding
domain. Cell.

[16] Bishop, C. M. ; Bishop, H. Deep learning: Foundations and concepts; Springer Nature, 2023.

[17] Jumper J., Evans R., Pritzel A., Green T., Figurnov M., Ronneberger O., Tunyasuvunakool K., Bates R., Žídek A., Potapenko A. (2021). Highly accurate protein structure prediction
with AlphaFold. nature.

[18] Lin Z., Akin H., Rao R., Hie B., Zhu Z., Lu W., Smetanin N., Verkuil R., Kabeli O., Shmueli Y. (2023). Evolutionary-scale prediction
of atomic-level protein structure with
a language model. Science.

[19] Krishna R., Wang J., Ahern W., Sturmfels P., Venkatesh P., Kalvet I., Lee G. R., Morey-Burrows F. S., Anishchenko I., Humphreys I. R. (2024). Generalized biomolecular
modeling and design with RoseTTAFold All-Atom. Science.

[20] Elofsson A. (2023). Progress at
protein structure prediction, as seen in CASP15. Curr. Opin. Struct. Biol..

[21] Jänes J., Beltrao P. (2024). Deep learning for protein structure
prediction and
design—progress and applications. Molecular
Systems Biology.

[22] Heinzinger M., Weissenow K., Sanchez J. G., Henkel A., Mirdita M., Steinegger M., Rost B. (2024). Bilingual language
model for protein
sequence and structure. NAR Genom. Bioinformat..

[23] Frazer J., Notin P., Dias M., Gomez A., Min J. K., Brock K., Gal Y., Marks D. S. (2021). Disease
variant
prediction with deep generative models of evolutionary data. Nature.

[24] Laine E., Karami Y., Carbone A. (2019). GEMME: a simple and
fast global epistatic
model predicting mutational effects. Molecular
biology and evolution.

[25] Sharma, A. ; Gitter, A. Exploring zero-shot structure-based protein fitness prediction. 2025, arXiv preprint arXiv:2504.16886.

[26] Ittisoponpisan S., Islam S. A., Khanna T., Alhuzimi E., David A., Sternberg M. J. (2019). Can predicted protein 3D structures
provide reliable
insights into whether missense variants are disease associated?. Journal of molecular biology.

[27] Zhang H., Xu M. S., Fan X., Chung W. K., Shen Y. (2022). Predicting
functional effect of missense variants using graph attention neural
networks. Nature Machine Intelligence.

[28] Tsishyn M., Cia G., Hermans P., Kwasigroch J., Rooman M., Pucci F. (2024). FiTMuSiC:
leveraging structural and (co) evolutionary data for protein fitness
prediction. Human Genomics.

[29] Wang, M. ; Nie, Z. ; He, Y. ; Vasilakos, A. V. ; Ren, Z. ProtFAD: Introducing function-aware domains as implicit modality towards protein function prediction. 2024, arXiv preprint arXiv:2405.15158.

[30] Nijkamp E., Ruffolo J. A., Weinstein E. N., Naik N., Madani A. (2023). Progen2: exploring
the boundaries of protein language models. Cell
systems.

[31] Bjerregaard A., Groth P. M., Hauberg S., Krogh A., Boomsma W. (2025). Foundation
models of protein sequences: A brief overview. Curr. Opin. Struct. Biol..

[32] Sledzieski S., Kshirsagar M., Baek M., Dodhia R., Lavista
Ferres J., Berger B. (2024). Democratizing protein language models
with parameter-efficient fine-tuning. Proc.
Natl. Acad. Sci. U. S. A..

[33] Glaser, M. ; Brägelmann, J. ESM-Effect: An Effective and Efficient Fine-Tuning Framework towards accurate prediction of Mutation’s Functional Effect. 2025, bioRxiv.

[34] Saadat A., Fellay J. (2025). Fine-tuning protein language models to understand the
functional impact of missense variants. Comput.
Struct. Biotechnol. J..

[35] Chu S. K., Narang K., Siegel J. B. (2024). Protein stability prediction by fine-tuning
a protein language model on a mega-scale dataset. PLoS computational biology.

[36] Gasperini M., Starita L., Shendure J. (2016). The power of multiplexed
functional
analysis of genetic variants. Nature protocols.

[37] Fowler D. M., Fields S. (2014). Deep mutational scanning:
a new style of protein science. Nat. Methods.

[38] Gelman S., Fahlberg S. A., Heinzelman P., Romero P. A., Gitter A. (2021). Neural networks
to learn protein sequence–function relationships from deep
mutational scanning data. Proc. Natl. Acad.
Sci. U. S. A..

[39] Tareen A., Kooshkbaghi M., Posfai A., Ireland W. T., McCandlish D. M., Kinney J. B. (2022). MAVE-NN: learning genotype-phenotype maps from multiplex
assays of variant effect. Genome Biol..

[40] Faure A. J., Lehner B. (2024). MoCHI: neural networks to fit interpretable
models
and quantify energies, energetic couplings, epistasis, and allostery
from deep mutational scanning data. Genome Biol..

[41] Chen C., Boorla V. S., Banerjee D., Chowdhury R., Cavener V. S., Nissly R. H., Gontu A., Boyle N. R., Vandegrift K., Nair M. S. (2021). Computational prediction
of the effect of amino acid changes on the binding affinity between
SARS-CoV-2 spike RBD and human ACE2. Proc. Natl.
Acad. Sci. U. S. A..

[42] Han J., Liu T., Zhang X., Yang Y., Shi Y., Li J., Ma M., Zhu W., Gong L., Xu Z. (2022). D3AI-Spike: A deep
learning platform for predicting binding affinity between SARS-CoV-2
spike receptor binding domain with multiple amino acid mutations and
human angiotensin-converting enzyme 2. Computers
in Biology and Medicine.

[43] Obermeyer F., Jankowiak M., Barkas N., Schaffner S. F., Pyle J. D., Yurkovetskiy L., Bosso M., Park D. J., Babadi M., MacInnis B. L. (2022). Analysis of 6.4 million
SARS-CoV-2 genomes identifies mutations associated with fitness. Science.

[44] Wang B., Gamazon E. R. (2022). Modeling mutational effects on biochemical phenotypes
using convolutional neural networks: application to SARS-CoV-2. iScience.

[45] Wang G., Liu X., Wang K., Gao Y., Li G., Baptista-Hon D. T., Yang X. H., Xue K., Tai W. H., Jiang Z. (2023). Deep-learning-enabled
protein–protein interaction analysis
for prediction of SARS-CoV-2 infectivity and variant evolution. Nature Medicine.

[46] Chen J., Woldring D. R., Huang F., Huang X., Wei G.-W. (2023). Topological
deep learning based deep mutational scanning. Computers in biology and medicine.

[47] Zhou B., Zhou H., Zhang X., Xu X., Chai Y., Zheng Z., Kot A. C., Zhou Z. (2023). TEMPO: A transformer-based
mutation prediction framework for SARS-CoV-2 evolution. Computers in Biology and Medicine.

[48] Wang D., Huot M., Mohanty V., Shakhnovich E. (2024). Biophysical
principles predict fitness of SARS-CoV-2 variants. Biophys. J..

[49] Liu X., Nie Z., Si H., Shen X., Liu Y., Huang X., Dong T., Xu F., Ren Z., Zhou P. (2025). Generative prediction
of real-world prevalent SARS-CoV-2 mutation
with in silico virus evolution. Brief. Bioinformat..

[50] Huot M., Wang D., Liu J., Shakhnovich E. I. (2025). Predicting
high-fitness viral protein variants with Bayesian active learning
and biophysics. Proc. Natl. Acad. Sci. U. S.
A..

[51] Xia H., Wei D., Guo Z., Chung L. W. (2025). Machine Learning
on the Impacts of
Mutations in the SARS-CoV-2 Spike RBD on Binding Affinity to Human
ACE2 Based on Deep Mutational Scanning Data. Biochemistry.

[52] Thadani N. N., Gurev S., Notin P., Youssef N., Rollins N. J., Ritter D., Sander C., Gal Y., Marks D. S. (2023). Learning
from prepandemic data to forecast viral escape. Nature.

[53] Elnaggar A., Heinzinger M., Dallago C., Rehawi G., Wang Y., Jones L., Gibbs T., Feher T., Angerer C., Steinegger M. (2022). Prottrans: Toward understanding the language
of life through self-supervised learning. IEEE
transactions on pattern analysis and machine intelligence.

[54] Gilmer, J. ; Schoenholz, S. S. ; Riley, P. F. ; Vinyals, O. ; Dahl, G. E. Neural message passing for quantum chemistry. In International conference on machine learning; PMLR, 2017; pp 1263–1272.

[55] Bronstein, M. M. ; Bruna, J. ; Cohen, T. ; Veličković, P. Geometric deep learning: Grids, groups, graphs, geodesics, and gauges. 2021, arXiv preprint arXiv:2104.13478.

[56] Vaswani, A. ; Shazeer, N. ; Parmar, N. ; Uszkoreit, J. ; Jones, L. ; Gomez, A. N. ; Kaiser, Ł. ; Polosukhin, I. Attention is all you need. In Advances in neural information processing systems; NIPS, 2017.

[57] Friedman J.
H. (2001). Greedy
function approximation: a gradient boosting machine. Ann. Statistics.

[58] Friedman J. H. (2002). Stochastic
gradient boosting. Computational statistics
& data analysis.

[59] Vinayak, R. K. ; Gilad-Bachrach, R. Dart: Dropouts meet multiple additive regression trees. In Artificial Intelligence and Statistics; JMLR, 2015; pp 489–497.

[60] Pak M. A., Markhieva K. A., Novikova M. S., Petrov D. S., Vorobyev I. S., Maksimova E. S., Kondrashov F. A., Ivankov D. N. (2023). Using AlphaFold
to predict the impact of single mutations on protein stability and
function. PLoS One.

[61] Buel G. R., Walters K. J. (2022). Can AlphaFold2 predict
the impact of missense mutations
on structure?. Nature structural & molecular
biology.

[62] Watson J. L., Juergens D., Bennett N. R., Trippe B. L., Yim J., Eisenach H. E., Ahern W., Borst A. J., Ragotte R. J., Milles L. F. (2023). De novo design of protein structure and function
with RFdiffusion. Nature.

[63] Michael R., Kæstel-Hansen J., Mo̷rch Groth P., Bartels S., Salomon J., Tian P., Hatzakis N. S., Boomsma W. (2024). A systematic analysis
of regression models for protein engineering. PLOS Comput. Biol..

[64] Livesey B. J., Badonyi M., Dias M., Frazer J., Kumar S., Lindorff-Larsen K., McCandlish D. M., Orenbuch R., Shearer C. A., Muffley L. (2025). Guidelines for releasing a variant effect predictor. Genome Biol..

[65] Organization, W. H . Updated working definitions and primary actions for SARSCOV2 variants. 2024. https://www.who.int/publications/m/item/updated-working-definitions-and-primary-actions-for--sars-cov-2-variants.

[66] Otwinowski J., McCandlish D. M., Plotkin J. B. (2018). Inferring the shape of global epistasis. Proc. Natl. Acad. Sci. U. S. A..

[67] Schulze, T. K. ; Blaabjerg, L. M. ; Cagiada, M. ; Lindorff-Larsen, K. Supervised learning of protein variant effects across large-scale mutagenesis datasets. 2025, bioRxiv. 10.1002/pro.70526 PMC12967566 41795207

[68] Geyer C. J. (1992). Practical
markov chain monte carlo. Statistical Sci..

[69] Jaynes E. T. (1957). Information
theory and statistical mechanics. Physical review.

[70] Jaynes, E. T. Probability theory: The logic of science; Cambridge University Press, 2003.

[71] Pitera J. W., Chodera J. D. (2012). On the use of experimental observations to bias simulated
ensembles. J. Chem. Theory Comput..

[72] Roux B., Weare J. (2013). On the statistical
equivalence of restrained-ensemble simulations
with the maximum entropy method. J. Chem. Phys..

[73] Amirkulova D. B., White A. D. (2019). Recent advances in maximum entropy biasing techniques
for molecular dynamics. Mol. Simul..

[74] Sztain T., Ahn S.-H., Bogetti A. T., Casalino L., Goldsmith J. A., Seitz E., McCool R. S., Kearns F. L., Acosta-Reyes F., Maji S. (2021). A glycan
gate controls opening of the SARS-CoV-2 spike
protein. Nature Chem..

[75] Mannar D., Saville J. W., Zhu X., Srivastava S. S., Berezuk A. M., Zhou S., Tuttle K. S., Kim A., Li W., Dimitrov D. S. (2021). Structural analysis
of receptor binding
domain mutations in SARS-CoV-2 variants of concern that modulate ACE2
and antibody binding. Cell Rep..

[76] Mannar D., Saville J. W., Sun Z., Zhu X., Marti M. M., Srivastava S. S., Berezuk A. M., Zhou S., Tuttle K. S., Sobolewski M. D. (2022). SARS-CoV-2 variants
of concern: spike protein
mutational analysis and epitope for broad neutralization. Nat. Commun..

[77] Mannar D., Saville J. W., Zhu X., Srivastava S. S., Berezuk A. M., Tuttle K. S., Marquez A. C., Sekirov I., Subramaniam S. (2022). SARS-CoV-2 Omicron variant: Antibody
evasion and cryo-EM
structure of spike protein–ACE2 complex. Science.

[78] Dommer A., Casalino L., Kearns F., Rosenfeld M., Wauer N., Ahn S.-H., Russo J., Oliveira S., Morris C., Bogetti A. (2023). # COVIDisAirborne: AI-enabled
multiscale computational microscopy of delta SARS-CoV-2 in a respiratory
aerosol. International Journal of High Performance
Computing Applications.

[79] Ni D., Turelli P., Beckert B., Nazarov S., Uchikawa E., Myasnikov A., Pojer F., Trono D., Stahlberg H., Lau K. (2023). Cryo-EM structures and binding of mouse and human ACE2 to SARS-CoV-2
variants of concern indicate that mutations enabling immune escape
could expand host range. PLoS pathogens.

[80] Hadfield J., Megill C., Bell S. M., Huddleston J., Potter B., Callender C., Sagulenko P., Bedford T., Neher R. A. (2018). Nextstrain: real-time
tracking of
pathogen evolution. Bioinformatics.

[81] McInnes, L. ; Healy, J. ; Melville, J. Umap: Uniform manifold approximation and projection for dimension reduction. 2018, arXiv preprint arXiv:1802.03426.

[82] Detlefsen N. S., Hauberg S., Boomsma W. (2022). Learning meaningful representations
of protein sequences. Nat. Commun..

[83] Ziegler C., Martin J., Sinner C., Morcos F. (2023). Latent generative
landscapes
as maps of functional diversity in protein sequence space. Nat. Commun..

[84] Ravuri, A. ; Lawrence, N. D. Towards one model for classical dimensionality reduction: A probabilistic perspective on umap and t-sne. 2024, arXiv preprint arXiv:2405.17412.

[85] Paszke, A. ; Gross, S. ; Massa, F. ; Lerer, A. ; Bradbury, J. ; Chanan, G. ; Killeen, T. ; Lin, Z. ; Gimelshein, N. ; Antiga, L. Pytorch: An imperative style, high-performance deep learning library. In Advances in neural information processing systems; NIPS, 2019.

[86] Agarap, A. F. Deep learning using rectified linear units (relu). 2018, arXiv preprint arXiv:1803.08375.

[87] Kingma, D. ; Adam, P. A method for stochastic optimization. 2014, arXiv preprint arXiv:1412.6980.

[88] Loshchilov, I. ; Hutter, F. Decoupled weight decay regularization. 2017, arXiv preprint arXiv:1711.05101.

[89] Defazio, A. ; Yang, X. ; Mehta, H. ; Mishchenko, K. ; Khaled, A. ; Cutkosky, A. The Road Less Scheduled; NIPS, 2024.

[90] Pless D. D., Lennarz W. J. (1977). Enzymatic conversion of proteins to glycoproteins. Proc. Natl. Acad. Sci. U. S. A..

[91] Alley E. C., Khimulya G., Biswas S., AlQuraishi M., Church G. M. (2019). Unified rational protein engineering
with sequence-based
deep representation learning. Nat. Methods.

[92] Rao, R. ; Bhattacharya, N. ; Thomas, N. ; Duan, Y. ; Chen, P. ; Canny, J. ; Abbeel, P. ; Song, Y. Evaluating protein transfer learning with TAPE. In Advances in neural information processing systems; NIPS, 2019. PMC7774645 33390682

[93] Heinzinger M., Elnaggar A., Wang Y., Dallago C., Nechaev D., Matthes F., Rost B. (2019). Modeling aspects of
the language
of life through transfer-learning protein sequences. BMC Bioinformat..

[94] Rives A., Meier J., Sercu T., Goyal S., Lin Z., Liu J., Guo D., Ott M., Zitnick C. L., Ma J. (2021). Biological structure
and function emerge from scaling unsupervised
learning to 250 million protein sequences. Proc.
Natl. Acad. Sci. U. S. A..

[95] Fey, M. ; Lenssen, J. E. Fast Graph Representation Learning with PyTorch Geometric. In ICLR Workshop on Representation Learning on Graphs and Manifolds; ICLR, 2019.

[96] Fey, M. ; Sunil, J. ; Nitta, A. ; Puri, R. ; Shah, M. ; Stojanovič, B. Y. ; Barghi, A. ; Kocijan, V. ; Zhang, Z. ; He, X. ; Lenssen, J. E. ; Leskovec, J. Jure PyG 2.0: Scalable Learning on Real World Graphs; KGL, 2025.

[97] Barros E. P., Casalino L., Gaieb Z., Dommer A. C., Wang Y., Fallon L., Raguette L., Belfon K., Simmerling C., Amaro R. E. (2021). The flexibility of ACE2 in the context of SARS-CoV-2
infection. Biophysical journal.

[98] Roe D. R., Cheatham T. E. (2013). PTRAJ and CPPTRAJ: software for processing
and analysis of molecular dynamics trajectory data. J. Chem. Theory Comput..

[99] McGibbon R. T., Beauchamp K. A., Harrigan M. P., Klein C., Swails J. M., Hernández C. X., Schwantes C. R., Wang L.-P., Lane T. J., Pande V. S. (2015). MDTraj: a modern open library for the analysis of molecular
dynamics trajectories. Biophysical journal.

[100] Devlin, J. ; Chang, M.-W. ; Lee, K. ; Toutanova, K. Bert: Pre-training of deep bidirectional transformers for language understanding. In Proceedings of the 2019 conference of the North American chapter of the association for computational linguistics: human language technologies, volume 1 (long and short papers); ACL, 2019; pp 4171–4186.

[101] Hendrycks, D. Gaussian Error Linear Units (Gelus). 2016, arXiv preprint arXiv:1606.08415.

[102] Ramachandran, P. ; Zoph, B. ; Le, Q. V. Swish: a self-gated activation function. 2017, arXiv preprint arXiv:1710.05941.

[103] Elfwing S., Uchibe E., Doya K. (2018). Sigmoid-weighted
linear
units for neural network function approximation in reinforcement learning. Neural networks.

[104] Xiong, R. ; Yang, Y. ; He, D. ; Zheng, K. ; Zheng, S. ; Xing, C. ; Zhang, H. ; Lan, Y. ; Wang, L. ; Liu, T. On layer normalization in the transformer architecture. In International conference on machine learning; PMLR, 2020; pp 10524–10533.

[105] Ke, G. ; Meng, Q. ; Finley, T. ; Wang, T. ; Chen, W. ; Ma, W. ; Ye, Q. ; Liu, T.-Y. Lightgbm: A highly efficient gradient boosting decision tree. In Advances in neural information processing systems; NIPS, 2017.

[106] Clevert, D.-A. ; Unterthiner, T. ; Hochreiter, S. Fast and accurate deep network learning by exponential linear units (elus). 2015, arXiv preprint arXiv:1511.07289.

[107] Hastings W. K. (1970). Monte Carlo sampling methods using
Markov chains and
their applications. Biometrika.

[108] Sayers E. W., Beck J., Bolton E. E., Brister J. R., Chan J., Connor R., Feldgarden M., Fine A. M., Funk K., Hoffman J. (2025). Database
resources of the National Center for Biotechnology Information in
2025. Nucleic acids research.

[109] Wu F., Zhao S., Yu B., Chen Y.-M., Wang W., Song Z.-G., Hu Y., Tao Z.-W., Tian J.-H., Pei Y.-Y. (2020). A new
coronavirus associated with human respiratory
disease in China. Nature.

[110] Cock P. J., Antao T., Chang J. T., Chapman B. A., Cox C. J., Dalke A., Friedberg I., Hamelryck T., Kauff F., Wilczynski B. (2009). Biopython: freely available Python tools for computational molecular
biology and bioinformatics. Bioinformatics.

