# Supplementary material for: Machine Learning-Driven Simulations of the SARS-CoV‑2 Fitness Landscape from Deep Mutational Scanning Experiments
Source: J Chem Inf Model. 2026 May 6;66(10):5721–35. doi: 10.1021/acs.jcim.6c00332 (PMC13213839; doi:10.1021/acs.jcim.6c00332)
Supplement: Supplementary file 1 [file ci6c00332_si_001.pdf]

Supporting Information: Machine Learning Driven Simulations of the SARS-CoV-2 Fitness Landscape from Deep Mutational Scanning Experiments

Aleksander E. P. Durumeric,[*,†,‡] Sean McCarty,[¶] Jay Smith,[¶] Jonas Köhler,[†] Katarina Elez,[†] Lluís Raich,[†] Patricia A. Suriana,[†,§] and Terra Sztain[*,†,¶]

†*Department of Mathematics and Computer Science, Freie Universität Berlin, Arnimallee 12, 14195 Berlin, Germany*

‡*Institute for Theoretical Physics, Heidelberg University, 69120 Heidelberg, Germany*

¶*Department of Medicinal Chemistry, University of Michigan, 428 Church St, Ann Arbor, MI 48109, United States*

§*Department of Computer Science, Stanford University, 353 Jane Stanford Way, Stanford, CA 94305, United States*

E-mail: aleksander.durumeric@fu-berlin.de; tsztain@umich.edu

Contents

I. Supplementary Methods

Data Generation

Network Training

Stopping Criteria and Evaluation

Loss Annealing

Tuning Heads

Performance Plateau

Input Representations

II. Supplementary Tables

Table S1. Optimal hyperparameters from one-hot MLP grid search on individual experiments.

Table S2. Parameter set explored for T5 MLPs used in this study.

Table S3. Scanned Transformer values for the original WT dataset.

Table S4. MCMC parameter influence on hotspot and VOC prediction.

Table S5. Summary of DMS datasets.

III. Supplementary Figures

Figure S1. Performance across multiple random splits of the original dataset.

Figure S2. Mutation count hold out splits.

Figure S3. Comparison of extrapolative performance of onehot and T5 with original and BA.1 datasets.

Figure S4. Linear calibration of predictions.

Figure S5. Optimization of MCMC simulation.

Figure S6. VOC predictions with DMS training data using $\Delta \log K_{\mathrm{D}}$ score thresholds.

Figure S7. Sequence profile of three MCMC replicates.

Figure S8. Top 20 residue positions identified.

Figure S9. Top 20 mutations with denoised score threshold.

Figure S10. Sequence profile of training data.

Figure S11. Top 20 mutations and positions colored by the 12 mutations appearing after BA.2.

Figure S12. Sequence profiles of the 12 mutations appearing after BA.2.

Figure S13. Top 20 mutations, positions, and sequence profiles from simulations centered around the BA.1 sequence.

Figure S14. UMAP of one-hot encoded sequences trained only on simulation and VOC.

I. Supplementary Methods

Data Generation

All DMS datasets were obtained from studies carried out by Bloom and colleagues. The original library initiated from the WT RBD sequence is described in ref 6. Briefly, two independent mutagenesis replicates were carried out using mutagenic primers with degenerate NNS codons tiling across the SARS-CoV-2 RBD sequence from residues 331-531 of the Wuhan-Hu-1 reference GenBank MN908947. A C-terminal fluorescent label was added to each RBD for quantification via fluorescence-activated cell sorting (FACS). RBDs were expressed in a yeast surface display system, and titrated with a range of fluorescently labeled ACE2 concentrations to calculate dissociation constants. Constants are reported as $\log K_{\mathrm{D}}$,app based on monomeric subunit concentrations of the dimeric ACE2 used. FACS-sorted libraries were analyzed with extensive quality control measures as described in ref 6. Briefly, libraries were sorted across 16 ACE2 concentrations with gating for singleton events and RBD-positive expression, and binding signal for each barcode was summarized as the mean fluorescence bin index. Titration curves were fit using weighted nonlinear least-squares regression, with weights derived from estimated variance as a function of per-sample cell count. Fits were constrained to the ACE2 concentration range of the titration. Variants with insufficient sampling depth were excluded. In addition, the worst-fitting 5% of titration

curves were removed based on normalized mean square residuals, with residuals scaled by the fitted response amplitude to avoid bias against low-signal variants. After filtering, $\log K_{\mathrm{D}}$,app estimates were retained for ∼75% of variants per library. These values were transformed to $\Delta \log K_{\mathrm{D,app}}$ relative to the WT, such that values above zero correspond to higher affinity than WT.

Network Training

This section outlines in more detail various aspects neural network training. Unless otherwise mentioned, each hyperparameter set was explored via grid search (e.g., 2048 MLP hyperparameter sets were investigated for one-hot embeddings on each dataset in Table S1).

Stopping Criteria and Evaluation

Training was performed using the mavenets `https://github.com/SztainLab/mavenets` package using the `train_tunable_model` function. Validation sets were selected via random sampling from the same experiments used for training. Mean squared error was used as both the training and validation loss. Early stopping was performed during training once no improvement in the validation loss was seen for 50 epochs. If early stopping did not occur, model training was stopped after 1000 epochs. Validation curves were then filtered using a rolling median of size 3 and the minimum value was recorded and used to rank the performance of each set of hyperparameters. The hyperparameters for the optimal model were then used to train a model three times with different random initializations. The best performing model of these three (as determined by validation set performance) was selected for evaluation on the held out test data.

Loss Annealing

When training models on multiple experiments, the loss used for training changed over the first 20 epochs. This change corresponded to shifting the coefficients of two linearly combined

losses. The first loss corresponded to the network prediction without adjustment via tuning heads, as where the second loss corresponds to the fully tuned prediction. The weights begin at 0.5 for both losses and progress to 0.0 and 1.0, respectively. This loss annealing strategy was chosen as it showed a stabilizing effect during early architectural exploration. The annealing schedule was not systematically tuned as part of the hyperparameter scanning procedure. Instead, it was chosen with the intuition that early encouragement for directly training the accuracy of the underlying network would reduce developing highly complex experimental tuning heads that could drive the network towards poorly performing training basins.

Tuning Heads

The tuning head architecture employed in the manuscript first feeds the non-experiment-specific prediction into a linear layer that expands dimensionality, applies an ELU, and then applies per-experiment linear layer to reduce the dimensionality to 1; the produced signal is then summed with the original prediction. An implementation of this approach is found in the `SharedFanTuner` class. Additionally, tuning heads comprised of linear models were also investigated; however, they did not result in competitive performance statistics (results not shown).

Performance Plateau

It is important to note that while the reported architectures are those which performed optimally on the validation set, many networks encountered during hyperparameter scanning performed similarly. For example, the top 10 scoring MLP architectures obtained when training on the original base dataset ranged in MSE performance between 0.232 and 0.236. The hyperparameters of these 10 candidates varied substantially, some with 2 hidden layers, some with 3, and with little pattern in layer width or weight decay. As a result, we avoid interpreting the optionally discovered hyperparameters in this work.

Input Representations

Different representations were used as input to different models. After initial experimentation, MLPs were trained on either one-hot embeddings or ProtT5 encodings. The the latter case, multiple strategies were used independently to reduce the size of the encodings to facilitate training on lower-end GPUs. First, encodings were mean pooled across the residue dimension. Second, PCA was performed on the sequence embedding obtained after concatenating the residue embeddings for a given sequence. Third, PCA was performed by considering every residue to be an independent sample; sequences were then embedded by projecting each residue using the same shared PCA matrix and concatenating the result. The PCA based approaches were found to do better when more dimensions were included without a clear saturation point; however, memory requirements (both CPU and GPU) limited the number of dimensions that were feasible. The largest per-residue PCA dimension under these constraints was 50 dimensions per residue, and the largest PCA dimension for the concatenated PCA was 500. Unless otherwise noted, all discussed results correspond to the third option as it showed the strongest performance. In all cases each feature dimension was standardized to have a mean of zero and standard deviation of 1 on the training data; this scaling was fixed and applied when evaluated the network on additional data. Transformers (not the PLM utilized for creating embeddings) were trained on a per-residue encoding summed with an encoding unique to each position in the sequence. MPNs were trained on one-hot embeddings as described in the main text.

II. Supplementary Tables

Table S1: Optimal hyperparameters from one-hot MLP grid search on individual experiments

model	layers	weight decay	learning rate
base WT	[16, 32, 16]	0.0001	0.0001
all data untuned	[8, 256, 32]	0.0001	0.0001
all data tuned	[8, 8, 8]	0.0005	0.0001
Wuhan - beta experiment	[16, 64]	0.005	0.0003
B.1.351	[16, 64]	0.0005	0.0003
N501Y	[8, 8, 8]	0.001	0.0003
E484K	[8, 32, 64]	0.001	0.0003
Wuhan - omicron experiment	[8, 8, 16]	0.005	0.0003
BA.1	[32, 8, 8]	0.0005	0.0001
BA.2	[256, 256, 256]	0.001	0.0001

Table S2: Parameter set explored for T5 MLPs used in this study. Networks with depths from 1-3 were tested, with layer widths applied independently.

Variable	Values
Hidden Layer Widths	128, 256, 512, 1028
Learning Rate	3e-4, 1e-4
Weight Decay	5e-3, 1e-3, 5e-4, 1e-4
Dropout	0, 0.1, 0.3, 0.5

Table S3: Scanned Transformer values for the original WT dataset. Optimal values shown in bold.

Variable	Values
Num. Block	1, 2, 3, 4, 5, **6**
Num. Heads	2, 4, **8**, 16
Embedding Size	16, 32, **64**
MHA Dropout	**0.05**, 0.1, 0.2
Block MLP Dropout	0.05, **0.1**, 0.2
Num. MLP Final Layers	0, **1**, 2, 3
Final MLP dropout	0.0, 0.05, **0.1**
Weight Decay	**5e-3**, 1e-3, 5e-4

Table S4: MCMC parameter influence on hotspot and VOC prediction. Combination of values used for analysis are shown in bold

β	C	max mutations from WT	hotspots	exact amino acid	same property
-5	0.95	9	5	7	5
**-10**	**0.95**	**9**	**5**	**11**	**5**
-15	0.95	9	7	10	5
-20	0.95	9	7	8	5
-10	0.5	9	5	10	5
-10	0.6	9	5	10	5
-10	0.7	9	5	10	5
-10	0.8	9	6	10	5
-10	0.9	9	5	10	5
-10	0.95	4	5	10	5
-10	0.95	15	5	11	5
-10	0.95	20	5	11	5

Table S5: Summary of DMS datasets.

Dataset	Strains	Size	Source	Data
Original	Wuhan Hu 1	105525	ref 6	link
B1.351	Wuhan Hu 1	13,449	ref 10	link
	N501Y	14682		
	E484K	11910		
	B1351 (Beta: K417N, E484K, N501Y)	12,722		
Omicron	Wuhan Hu 1	13,892	ref 11	link
	BA1	9,161		
	BA2	8,191		

III. Supplementary Figures

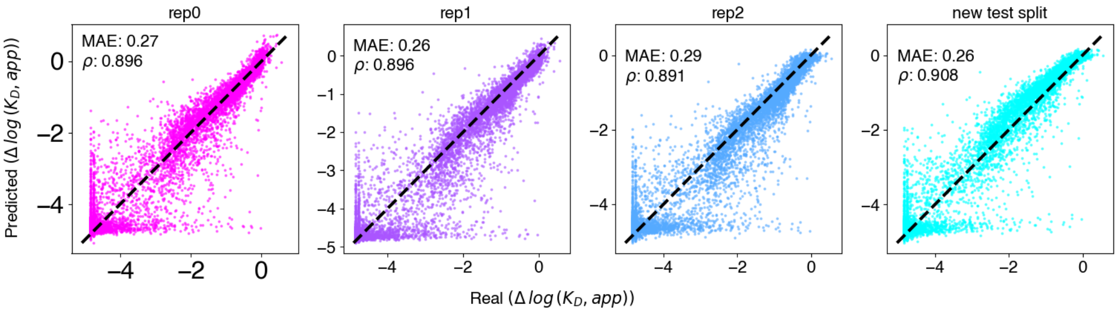

Figure S1: Performance across multiple random splits of the original dataset. These replicates were carried out in the exact same way as the initial split. A hyperparameter grid search was carried out for various MLPs with one-hot embeddings. The model with the best validation loss out of three replicate trainings was selected. The first three replicates show random re-splitting of the train and validation splits with a fixed test set, whereas the last shows performance on a complete re-splitting of all three sets.

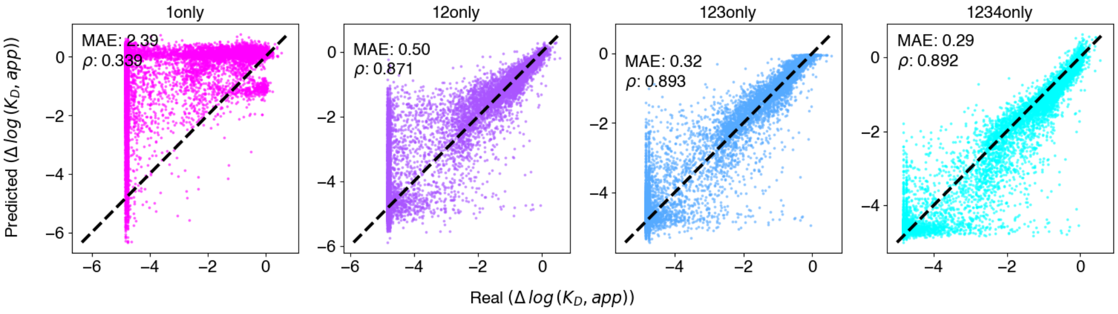

Figure S2: Mutation count hold out splits. Training and validation splits were limited to either point mutants or up to double, triple, or quadruple mutants. A hyperparameter grid search was carried out for various MLPs with one-hot embeddings. The model with the best validation loss out of three replicate trainings was selected evaluated the original test set.

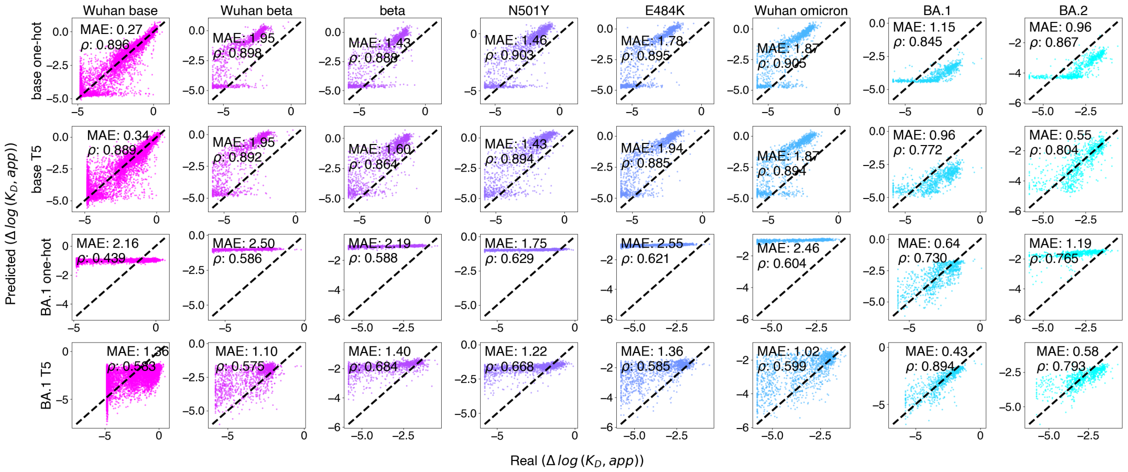

Figure S3: Comparison of extrapolative performance of onehot and T5 with original and BA.1 datasets. Notably the Spearman coefficient is higher for onehot than T5 for experiments not used in the training. The only case where T5 coefficient is larger than onehot is BA.1 predicting on BA.1, which is in distribution, not extrapolation.

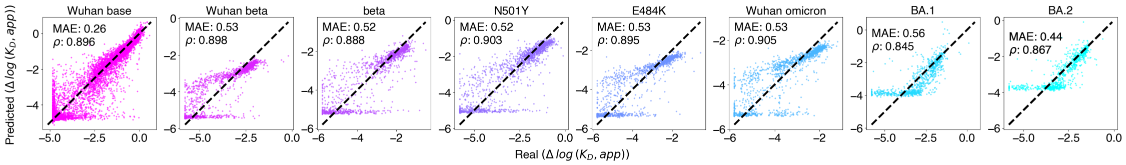

Figure S4: Linear calibration of predictions. Results from model trained on original dataset with one-hot encoding, extrapolating predictions to the additional datasets.

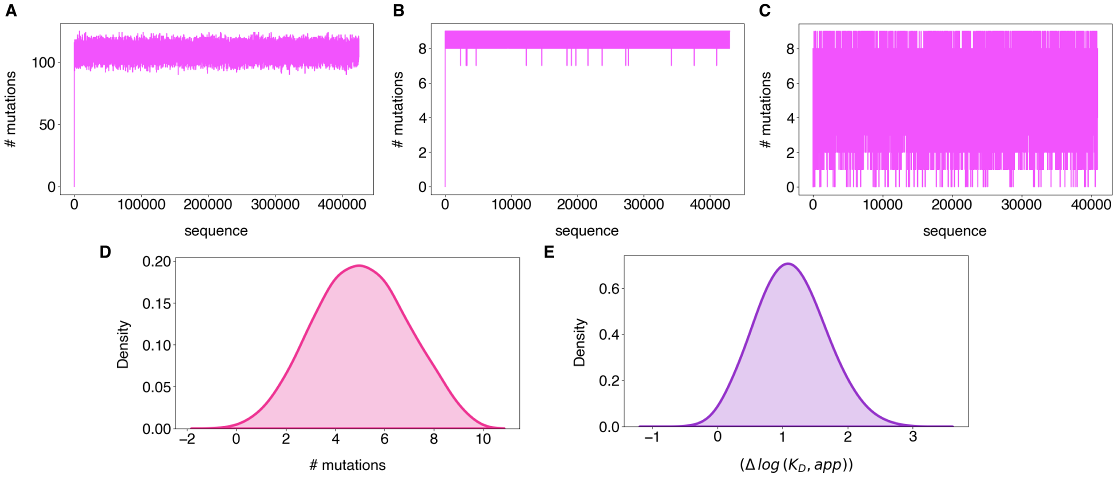

Figure S5: Optimization of MCMC simulation. A. Without any proposal bias. B. With maximum mutation count set. C. With proposal bias in Eq. 2 applied. D. Smoothed mutation distribution of simulation with optimal parameters E. Smoothed $\Delta \log K_{\mathrm{D}}$ distribution of simulation with optimal parameters.

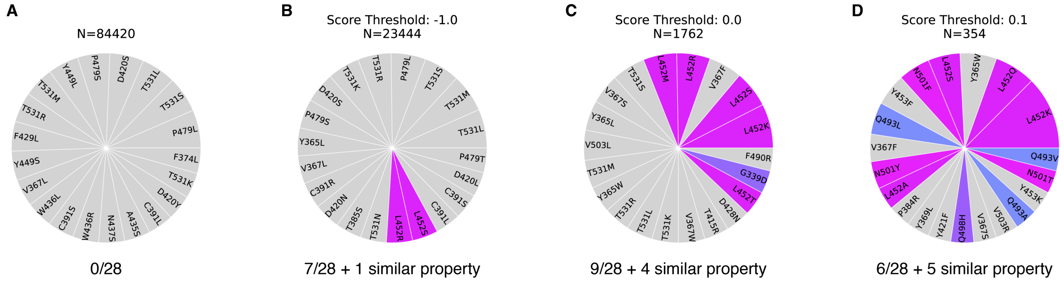

Figure S6: VOC predictions with DMS training data using $\Delta \log K_{\mathrm{D}}$ score thresholds. Score threshold and number of sequences which pass are labeled above each chart. Wedges are colored based on residue number, corresponding to those in the top 20 Genbank mutations. Number of VOC correctly predicted as the most frequent mutation at a given position are indicated below each chart.

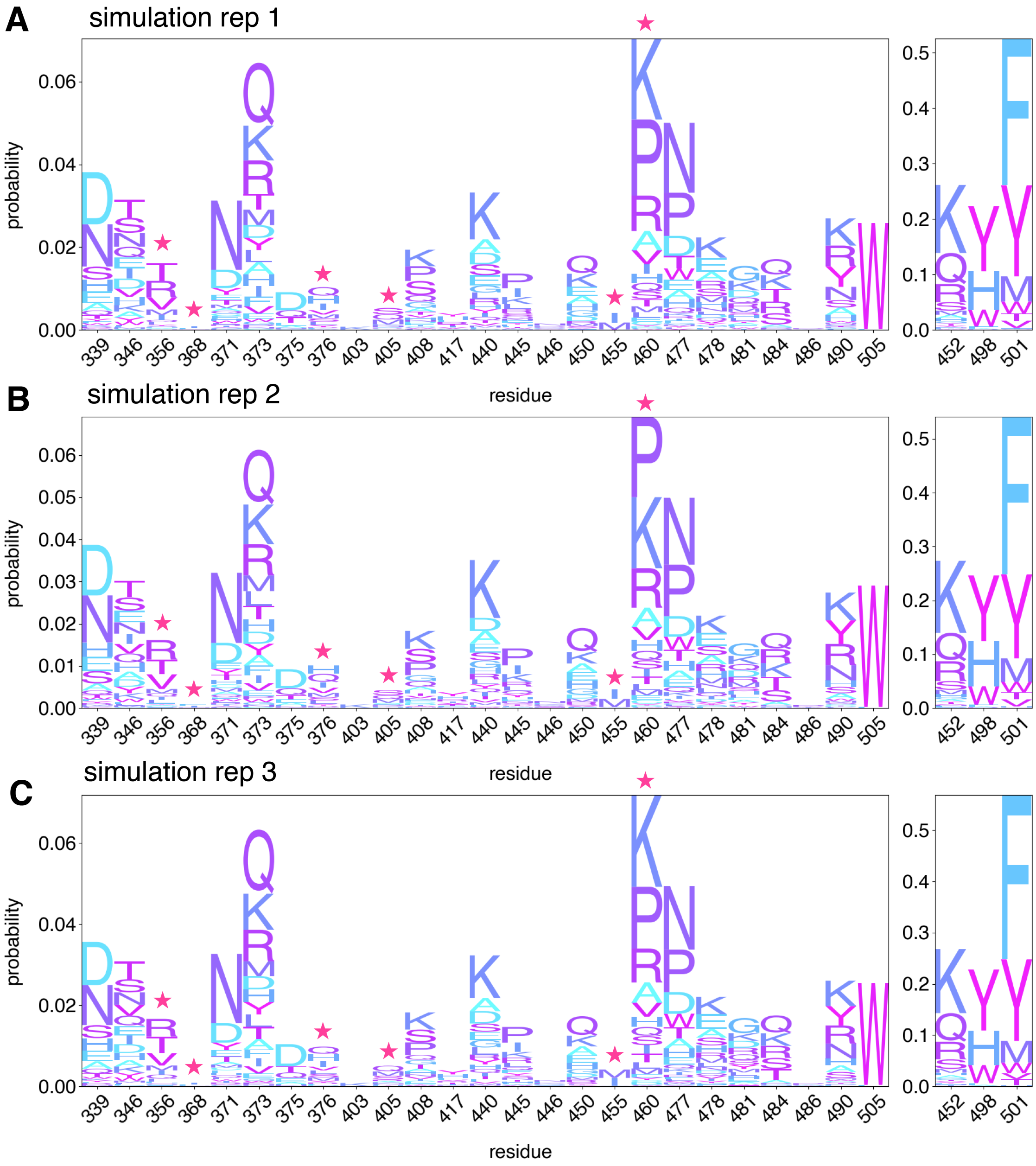

Figure S7: Sequence profile of three MCMC replicates. Residues with a difference in any replicate of most prevalent amino acid mutation are highlighted with a pink star.

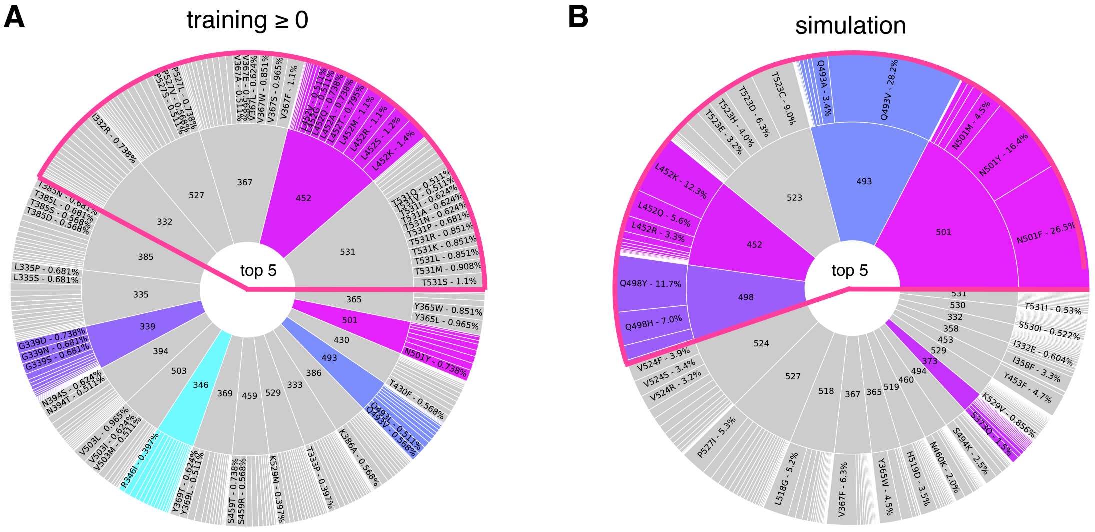

Figure S8: Top 20 residue positions identified. A. DMS training data using a $\Delta \log K_{\mathrm{D}}$ score threshold of 0 (better than WT) B. MCMC simulation. Wedges are colored based on residue number, corresponding to those in the top 20 Genbank mutations. The top 5 residue positions are highlighted with a pink outline. Sequence profile is shown as a nested pie chart with the inner layer corresponding to the residue position, and outer layer indicating which amino acid substitutions are most frequent at a given position.

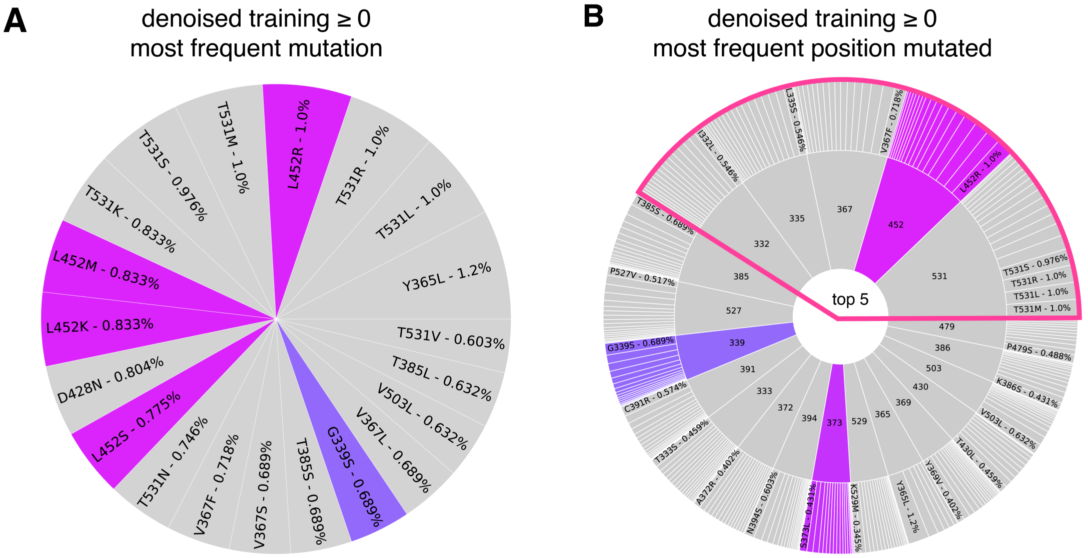

Figure S9: Top 20 mutations with denoised score threshold. A. Top 20 mutations overall, B. Top 20 positions that are mutated, regardless of which amino acid they are mutated to. Wedges are colored based on residue number, corresponding to those in the top 20 Genbank mutations. Outer ring slices indicate different amino acids with the most frequent substitution labeled.

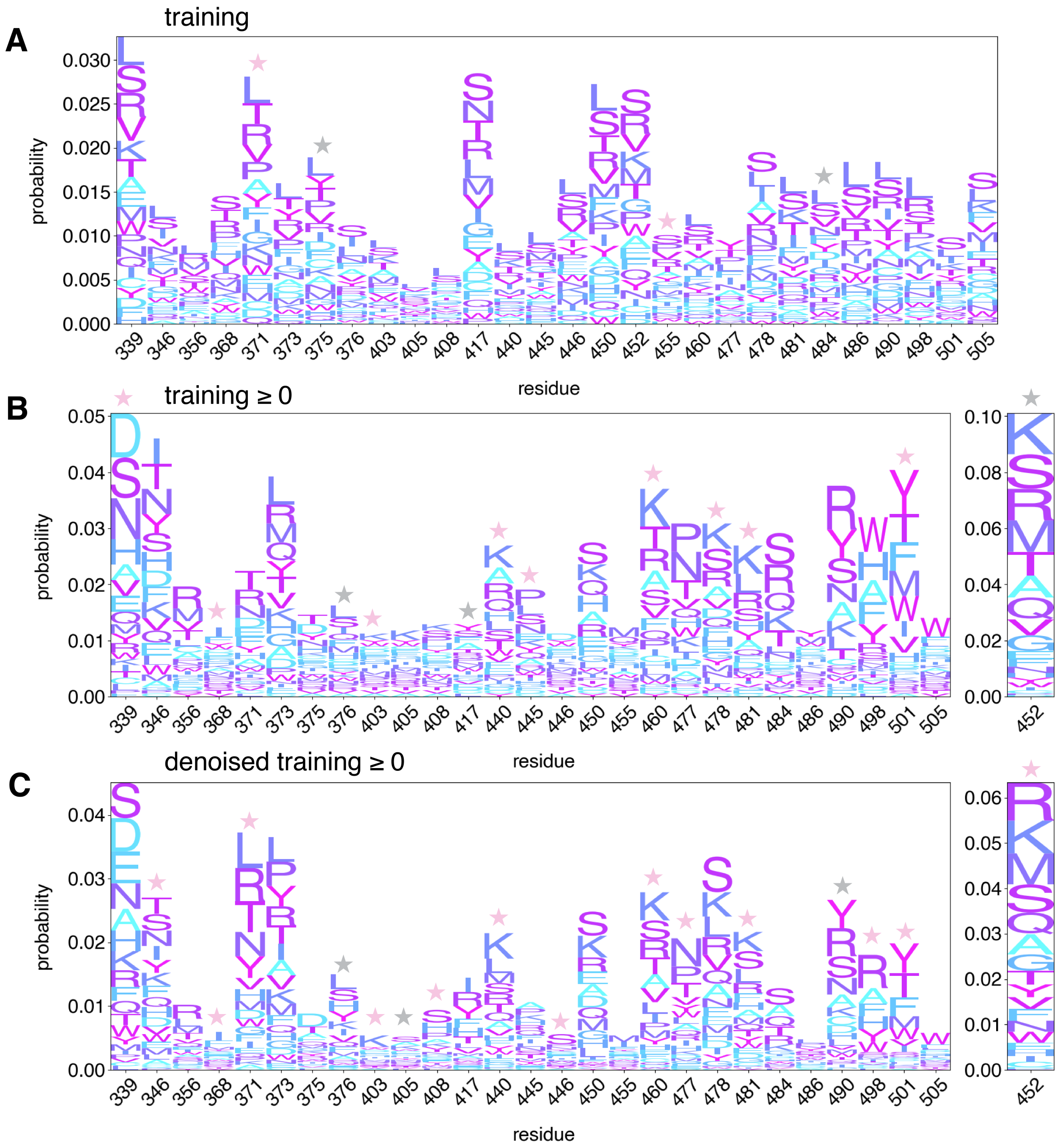

Figure S10: Sequence profile of training data. A. Without a score threshold B. With a score threshold of 0 (better than WT) and C. With a score threshold of 0, with scores recalculated from running sequences through the trained ML model. A pink star indicates the probable amino acid at a given position corresponds to a VOC mutation. A gray star indicates the most probable amino acid has the same physiochemical properties as the VOC mutation, such as polar, basic, and aromatic

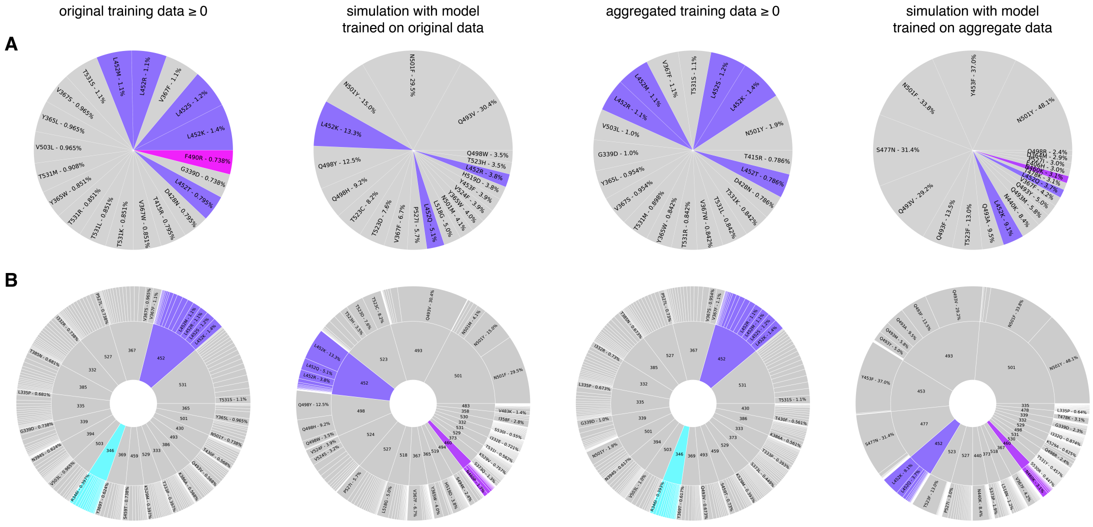

Figure S11: Top 20 mutations and positions colored by the 12 mutations appearing after BA.2. Wedges are colored based on residue number, corresponding to those in the top 20 Genbank mutations, only if they belong to the 12 residues with mutations after BA.2 (See **Figure S12** for the 12 residues considered). A. Top 20 mutations overall from the original training data with threshold, simulation from model trained on original data, aggregated training data from all 8 DMS libraries with threshold, and simulation from per-experiment tuned model trained on data from all 8 libraries. B. Top 20 position regardless of mutation for each. The outer ring slices indicating different amino acids, with the most frequent substitution labeled.

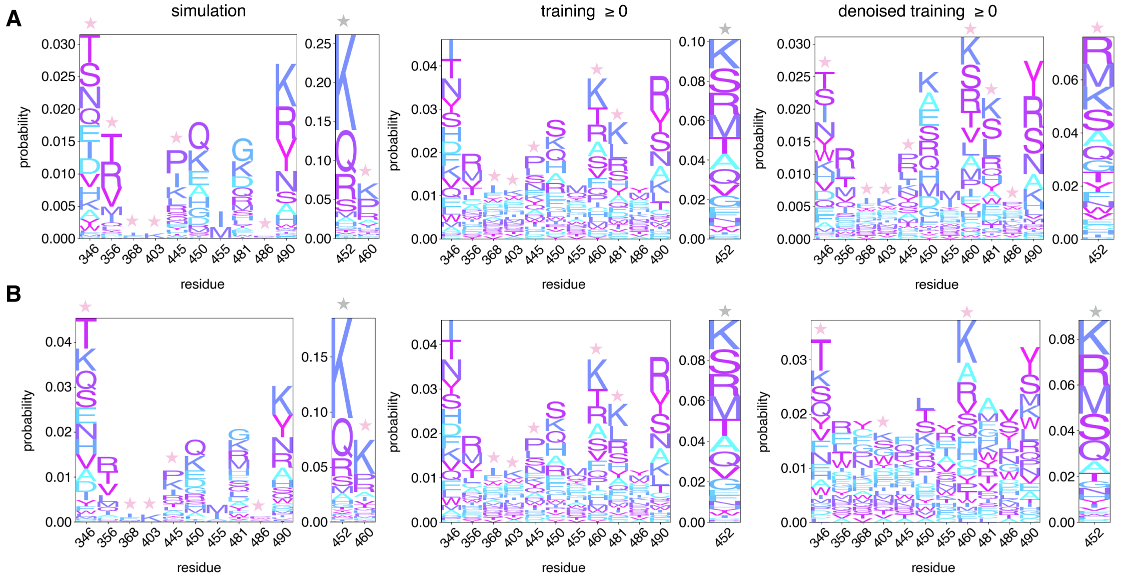

Figure S12: Sequence profiles of the 12 mutations appearing after BA.2. A. profiles from the simulation using model trained on the original data, the original training data with a $\Delta \log K_{\mathrm{D}}$ threshold of 0, and denoised training data, with the threshold recalculated after running the training data through the model trained on the original data set. B. Sequence profiles for each category in A. using the per-experiment tuned model and training data from aggregating all 8 DMS libraries. Note the sequence profiles of the training data with threshold of 0 for the original and aggregate data appear identical because only a small number of additional sequences from the new libraries passed the score threshold. Various threshold values were considered and a threshold of 0 was still the most predictive. A pink star indicates the probable amino acid at a given position corresponds to a VOC mutation. A gray star indicates the most probable amino acid has the same physiochemical properties as the VOC mutation, (ie. L452R predicted as K is also a mutation from non-polar to basic amino acid).

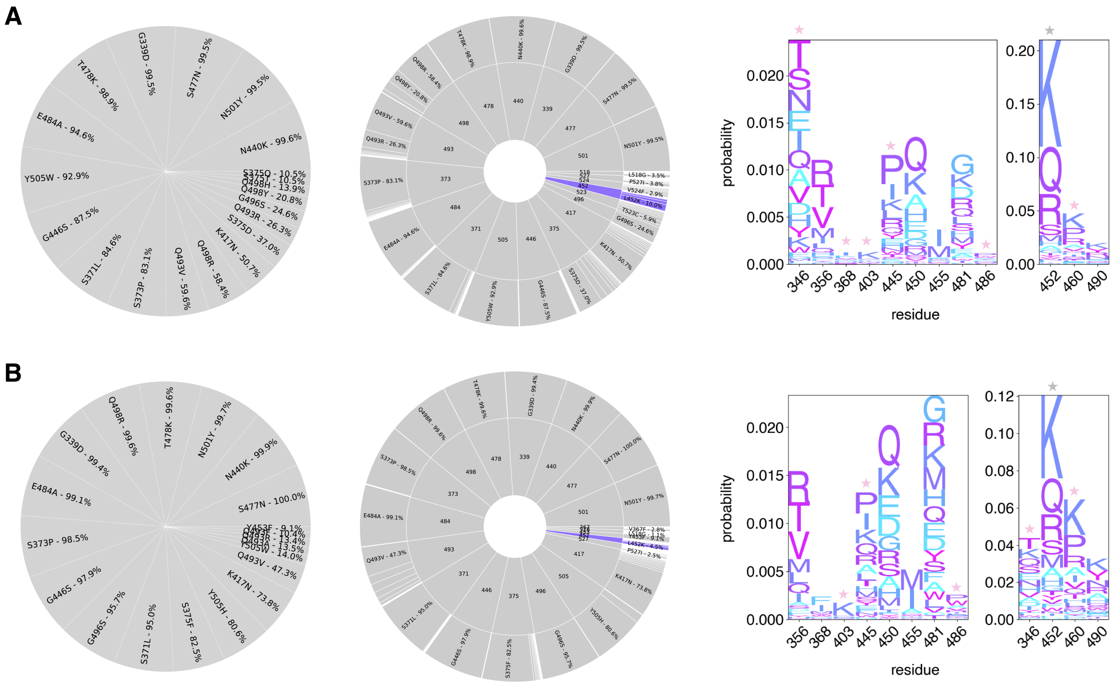

Figure S13: Top 20 mutations, positions, and sequence profiles from simulations centered around the BA.1 sequence. A. Simulations using model trained on original DMS data. B. Simulations using model trained on aggregated data with per-experiment tuning. Residues are colored in pie charts if they are in the list of 12 mutations appearing after BA.2. All charts follow the same formatting detailed in the previous figures.

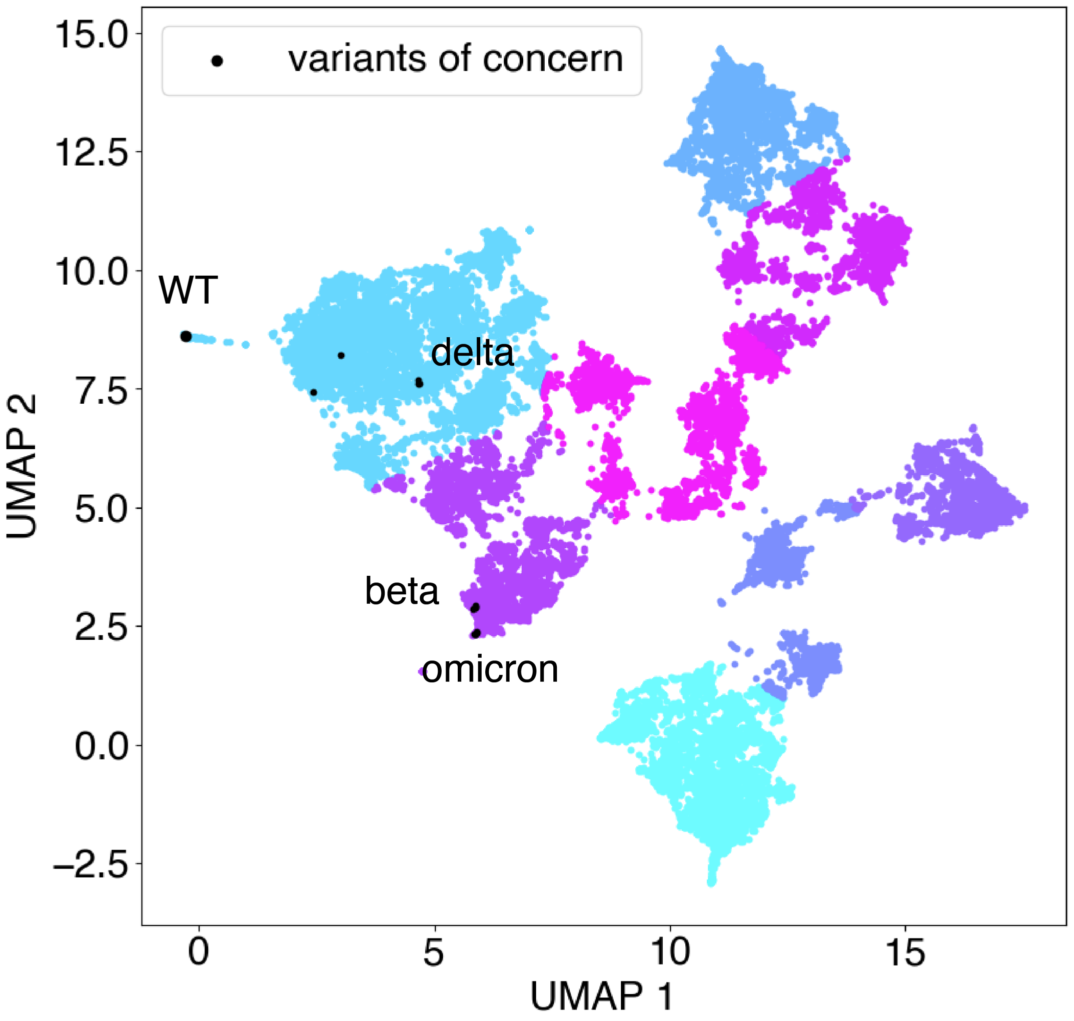

Figure S14: UMAP of one-hot encoded sequences trained only on simulation and VOC. Simulation results are from original WT-trained model. Colors are based on K-means clustering. VOCs from **Table 1** are plotted as black dots with select categories labeled.
